# PDGF enhances the protective effect of adipose stem cell-derived extracellular vesicles in a model of acute hindlimb ischemia

**DOI:** 10.1038/s41598-018-36143-3

**Published:** 2018-12-04

**Authors:** Tatiana Lopatina, Enrica Favaro, Cristina Grange, Massimo Cedrino, Andrea Ranghino, Sergio Occhipinti, Sofia Fallo, Fabrizio Buffolo, Daria A. Gaykalova, Maria M. Zanone, Renato Romagnoli, Giovanni Camussi

**Affiliations:** 1 Department of Medical Sciences, University of Turin, Turin, Italy; 2 2i3T, Società per La Gestione Dell’incubatore Di Imprese e Per Il Trasferimento Tecnologico Dell’Università degli Studi di Torino, Scarl., Molecular Biotechnology Center (MBC), Turin, Italy; 3 General Surgery 2U, Liver Transplantation Center, AOU Città della Salute e della Scienza di Torino, University of Turin, Turin, Italy; 4 Otolaryngology - Head and Neck Surgery, The Johns Hopkins University School of Medicine, 1550 Orleans street, Rm 5M06, CRBII, Baltimore, MD 21231 United States of America

## Abstract

We previously have shown that platelet-derived growth factor (PDGF) modulates the biological activity of extracellular vesicles released by adipose-derived mesenchymal stem cells (ASC-EVs). ASC-EVs may interact with blood and vessel cells by transferring proteins and nucleic acids and regulate their functions. In this study, we investigated immunomodulatory activity and protection from acute hindlimb ischemia of EVs released by PDGF-stimulated ASC (PDGF-EVs). PDGF treatment of ASC changed protein and RNA composition of released EVs by enhancing the expression of anti-inflammatory and immunomodulatory factors. *In vitro*, control EVs (cEVs) derived from non-stimulated ASC increased the secretion of both the IL-1b, IL-17, IFNγ, TNFα pro-inflammatory factors and the IL-10 anti-inflammatory factor, and enhanced the *in vitro* peripheral blood mononuclear cell (PBMC) adhesion on endothelium. In contrast, PDGF-EVs enhanced IL-10 secretion and induced TGF-β1 secretion by PBMC. Moreover, PDGF-EVs stimulated the formation of T regulatory cells. *In vivo*, PDGF-EVs protected muscle tissue from acute ischemia, reduced infiltration of inflammatory cells and increased T regulatory cell infiltration in respect to cEVs. Our results suggest that PDGF-EVs are enriched in anti-inflammatory and immunomodulatory factors and induced in PBMC an enhanced production of IL-10 and TGF-β1 resulting in protection of muscle from acute ischemia *in vivo*.

## Introduction

Extracellular vesicles (EVs) are intercellular shuttles that were found in almost all biological liquids, such as saliva, urine, liquor, amniotic liquid, peritoneal fluid, and plasma. EVs contain a specific set of lipids, proteins, and nucleic acids and can regulate the cell function and gene expression of the recipient cells^1–3^. Vessel cells, such as endothelial cells, smooth muscle cells, or mesenchymal stem cells, release their EVs in the bloodstream, where they interact with blood cells. Adipose-derived mesenchymal stem cells (ASC) are situated within the vessel wall and have a critical pro-angiogenic function. Previously we have shown that EVs secreted by ASC (ASC-EV) enhance the angiogenesis *in vitro* and *in vivo*^4^ and that platelet-derived growth factor b (PDGF) significantly enhances this pro-angiogenic action^5^. In general, PDGF plays an essential role in angiogenesis and tissue regeneration^6^. It is secreted either by platelets during vessel injury or by other cells, such as mesenchymal or endothelial cells^7^. Normally, PDGF is produced after the tissue injury, and its level decreases several days later^8^. During the inflammation, PDGF induces migration and proliferation of monocytes, fibroblasts and vascular smooth muscle cells, attracts monocytes to the site of the vascular injury, and limits pro-inflammatory events through the autocrine feedback inhibition of the platelet aggregation^9^. PDGF is also a strong pro-angiogenic factor^10^. ASC express the receptor for PDGFbb (PDGFRβ), which induces proliferation, migration, and angiogenic properties of ASC by up-regulating relevant paracrine factors^11^.

Angiogenesis, tissue repair and inflammation are closely related processes. In this study aimed to define the effect of ASC-derived EVs, released in response to PDGF stimulation (PDGF-EVs), in tissue regeneration and inflammation. For this purpose, we compared the *in vitro* effect of control EVs (cEVs) and PDGF-EVs on peripheral blood mononuclear cells (PBMC). We compared protein and RNA composition of PDGF-EVs and cEVs and the effect of PBMC stimulation by these EVs. In particular, the cytokine secretion, PBMC differentiation and activation, and PMBC adhesion to endothelium were evaluated. *In vivo*, we investigated the effects of PDGF-EVs and cEVs on injured muscles using a model of acute hindlimb ischemia in mice.

## Results

### Characterization of PDGF-EVs

**Figure 1:**
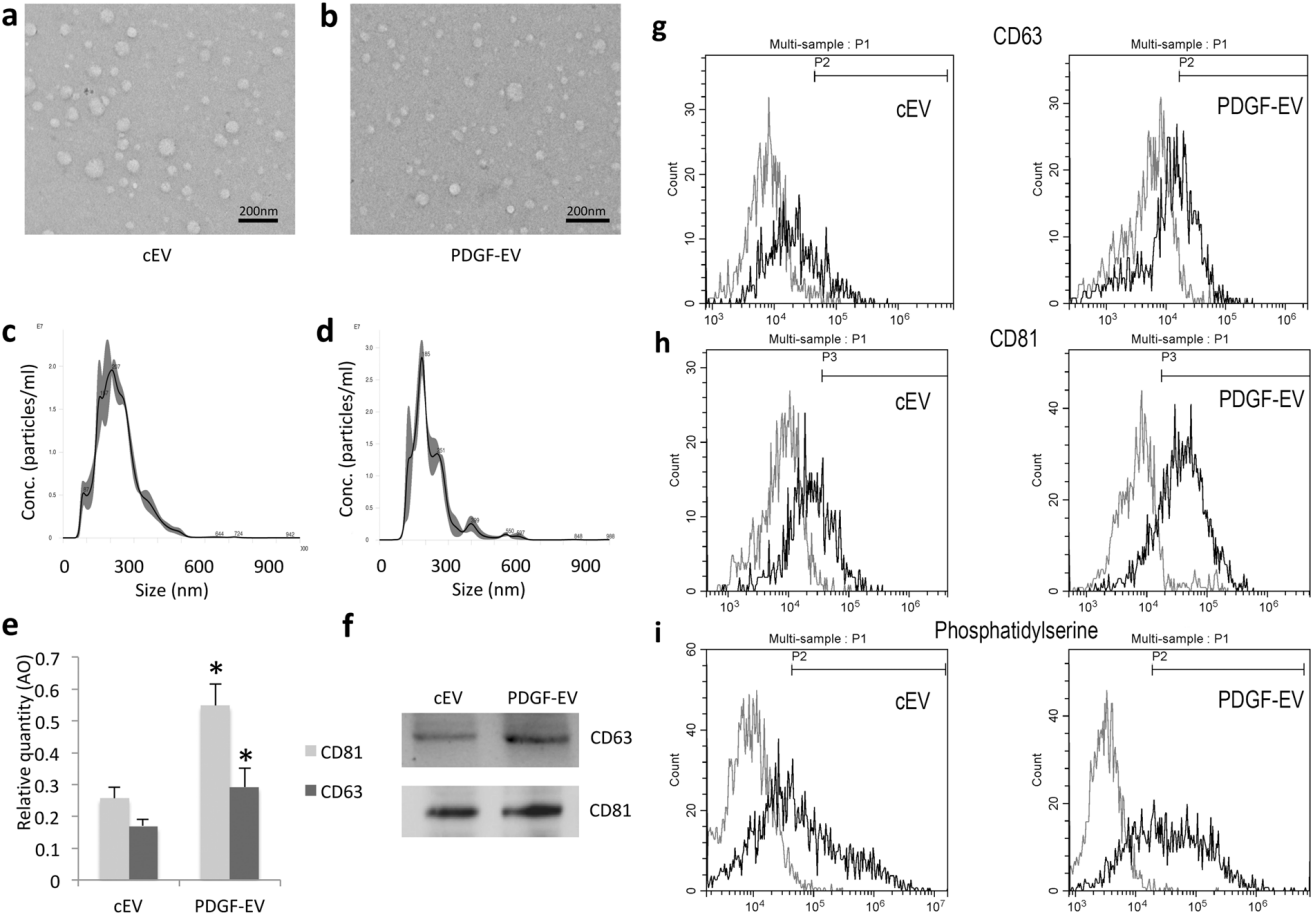
The characterization of ASC-derived EVs, collected after cell stimulation with PDGF. (**a**,**b**) The representative transmission electron microscopy images of cEVs (**a**) and PDGF-EVs (**b**) negatively stained with NanoVan (black line = 500 nm); (**c**,**d**) the representative diagram of cEVs (**c**) or PDGF-EVs (**d**) according to NTA; (**e**) the diagram of CD63 and CD81 concentration in cEVs and PDGF-EVs according to ELISA, (mean ± SEM, *p < 0.05 vs. cEVs, n = 6); (**f**) the representative western blot images of CD63 and CD81 expression in ASC-derived EVs, 10 μg of protein per lane; (**g**) the representative image of FACS analysis of CD63 expression on cEV (left) and PDGF-EV (right); (**h**) the representative image of FACS analysis of CD81 expression on cEV (left) and PDGF-EV (right); (**i**) the representative image of FACS analysis of phosphatidylserine expression on cEV (left) and PDGF-EV (right).

To validate our previous data^5^, we confirmed no difference in EV size (90 nm) and morphology between cEVs and PDGF-EVs according to electron microscopy and nanoparticle tracking analysis (NTA) (Fig. 1a–d). Similar to our previous result PDGF stimulation significantly enhanced EV release^5^ according to the expression of exosomal markers CD63 and CD81 validated by ELISA (Fig. 1e). FACS analysis confirmed that the PDGF-EV population was significantly enriched in CD63^+^ (15.3% ± 6.8 of cEVs, vs. 27.7% ± 14.3 of PDGF-EVs, p = 0.045, Fig. 1g), CD81^+^ (26.5% ± 8.9 of cEVs, vs. 64.9% ± 12.3 of PDGF-EVs, p = 0.039, Fig. 1h), and phosphatidylserine positive particles (47.5% ± 13.8 of cEVs, vs. 76.8% ± 9.8 of PDGF-EVs, p = 0.028, Fig. 1i).

The expression of CD47 (32.8% ± 16, Supplementary Fig. S1c), CD105 (62.2% ± 11.4, Supplementary Fig. S1d), HLA class I (37.6% ± 8, Supplementary Fig. S1e), HLA class II (0.8% ± 4, Supplementary Fig. S1f), ICAM (35% ± 14.4, Supplementary Fig. S1g), and VE-cadherin (52.9% ± 9.4, Supplementary Fig. S1h), was not statistically different even if an enhanced expression trend was observed in PDGF-EVs. PDGF did not change the expression of CD105, HLA, and VE-cadherin, which are markers of the cell of origin, and of ICAM and CD4, which are ubiquitously expressed molecules.

### PDGF-EVs contain a unique composition of proteins related to inflammation and tissue regeneration

Using protein array, we have evaluated the expression of 1,000 proteins in PDGF-EVs and their controls in two independent experiments. The only proteins consistently up- or down-regulated were considered differentially expressed. Thus, we found 65 proteins to be consistently up-regulated, 15 proteins to be consistently down-regulated in PDGF-EVs relative to their non-stimulated EV controls. We have also detected 228 proteins with an equal expression (RQ more than 0.5 and less than 2) between PDGF-EVs and cEV (Supplementary Table S1, Fig. S2).

The bioinformatic analysis of EV proteins revealed that both populations of EVs carried regulators of inflammation, but PDGF-EVs were significantly enriched with proteins implicated in immunomodulation such as TGFβ1, TRAIL, TROY, CCR7, CD71, CXCL11, LTBP, TLR4, TGFBR3, TNFSF8, TNFSF10, IL-23A, CD38, GLYPICAN 5, ADAMTS, and proteins involved in pro-regenerative process such as VEGFs, BDNF, FGF21. On the other hand, PDGF-EVs were diminished of Activin, TNFRSF13C, CD80, angiopoietin-like 2, and RIP1, known to promote inflammation (Supplementary Fig. S3b).

### PDGF-EVs contain a unique composition of RNA molecules

We performed PCR array analysis of long non-coding (lnc) RNA and microRNA in PDGF-EVs and cEVs. This analysis revealed that PDGF-EVs contained significantly more lncRNA MALAT1 (RQ 5.1, p = 0.013) a well-described anti-inflammatory^12^, pro-angiogenic^13^, and pro-regenerative regulator^12,14,15^.

MicroRNA analysis also revealed a significant difference in the expression of several miRNAs: PDGF-EVs carry significantly more miR-502 (RQ = 16.5, p = 0.042), miR-99a (RQ = 6.0, p = 0.03), miR-203 (RQ = 2.1 p = 0.017), miR-125 (RQ = 2.4, p = 0.031), and miR-195 (RQ = 2.4, p = 0.034), and were depleted of miR-1225 (RQ = 0.3, p = 0.05) and miR-1226 (RQ = 0.5, p = 0.005). Several microRNA were detected exclusively in cEVs or in PDGF-EVs (Supplementary Table S2). EV expression of MALAT1, miR-29a, miR-126-3p, miR-92a-3p, and miR-145-5p was independently confirmed by Real-time PCR (Supplementary Fig. S3). Bioinformatic analysis performed using DIANA TOOL mirPath V3^16^ revealed that PDGF-EVs carried miRNAs that potentially target TNFα (miR-551b-3p), inhibitors of TGF-β signaling pathway (miR-let7f-2–3p), and pseudoreceptor BAMBI, a negative regulator of TGF-β (miR-99a-3p). On the other hand, cEV carried miRNAs that potentially target precursor of TGF-β LTBP1 (miR-1, miR-541-3p, and miR-659-3p) and SMAD4 (miR-483-3p). Overall, PDGF-EVs contained miRNAs implicated in TGF signaling and TNF signaling, whereas cEVs overrepresented fatty acid metabolism pathways (Supplementary Table S3).

### PDGF-EVs possess anti-inflammatory properties

PDGF-stimulated and non-stimulated ASC-EVs contain distinct patterns of proteins and RNA molecules, relevant to inflammation. We, therefore, investigated the influence of these EVs on inflammatory cells *in vitro*.

**Figure 2:**
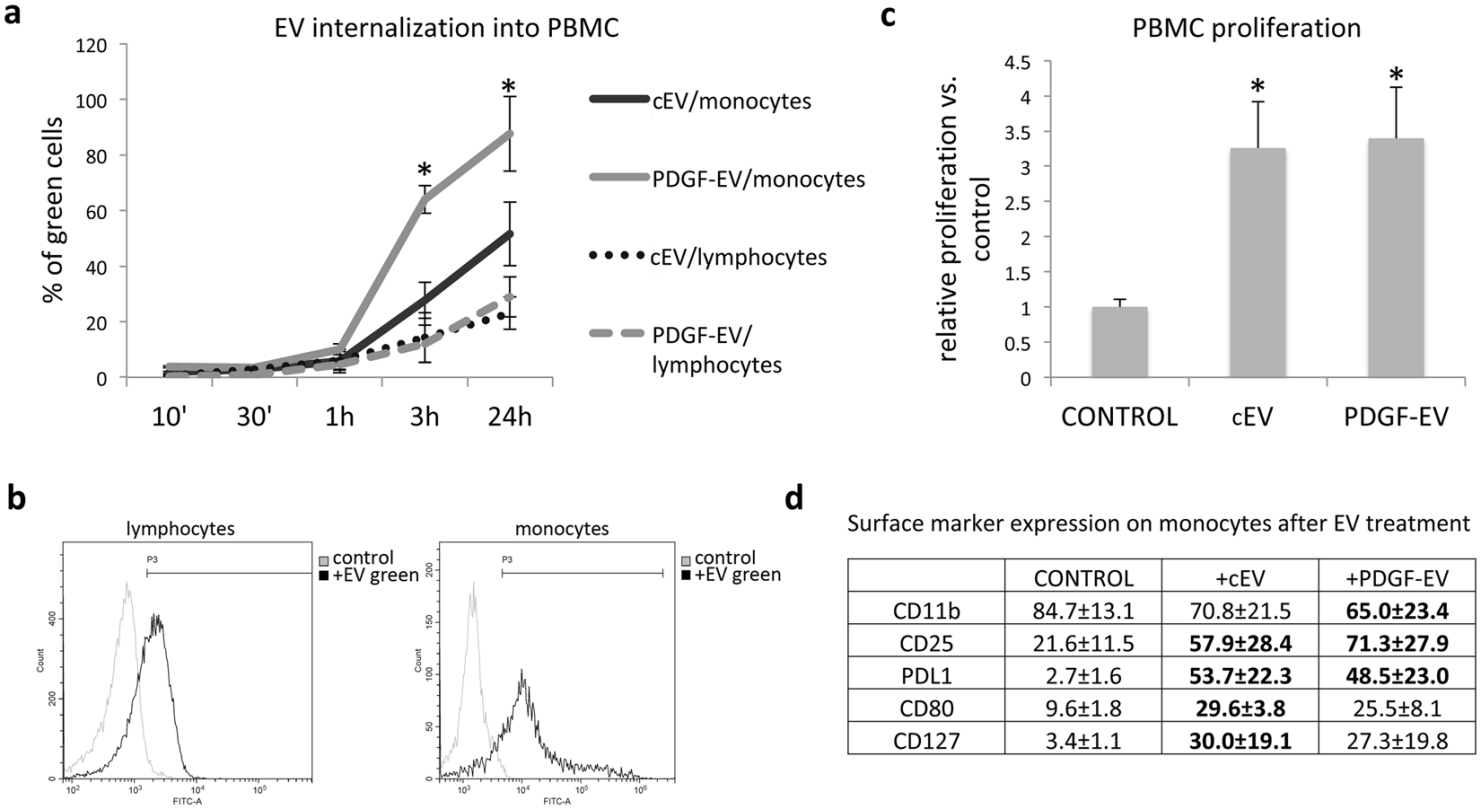
EV interaction with PBMC. (**a**) The diagram of EV internalization in monocyte and lymphocyte PBMC fractions during 24 hours. Total PBMC fractions isolated from healthy donors were stimulated with EVs labeled with PKH67GL green fluorescent cell linker. After the indicated time periods cells were analyzed by FACS, and the statistics was performed on monocyte and lymphocyte gates separately (mean ± SEM, *p < 0.05 vs. cEVs, n = 5); (**b**) the representative images of FACS analysis of PBMC incubated with PKH67GL labeled EVs during 24 hours. The left diagram shows the lymphocyte fraction; the right diagram shows the monocyte fraction. (**c**) The diagram of PBMC proliferation rate after stimulation with EVs, analyzed by BrdU ELISA (mean ± SEM, *p < 0.01 vs. control PBMC, n = 3); (**d**) the surface marker expression on monocytes after EV treatment (mean % ± SEM, in bold - p < 0.05 vs. control PBMC, n = 4).

Using FACS analysis, we evaluated the ability of PKH67GL-labeled EVs to be internalized by PBMC (Fig. 2b). As shown in Fig. 2a, EVs were preferentially internalized into the monocyte fraction of PBMC. Moreover, PDGF-EVs were internalized significantly faster into the monocytes than cEVs (Fig. 2a). Both types of EV stimulated PBMC proliferation (Fig. 2c) and increased the number of CD25^+^ cells (Fig. 2d). The number of CD8^+^, CD20^+^ or CD19^+^ did not change upon EV stimulation. Both types of EVs induced the expression of PDL1 on monocytes. PDGF-EV decreased the expression of CD11b, whereas, cEVs significantly enhanced the expression of the monocyte activation markers CD80 and CD127 (Fig. 2d).

To evaluate the effect of EVs on PBMC gene expression, we performed PCR array-based gene expression analysis of 84 genes involved in inflammation. The cEVs led to significant up-regulation of 10 pro-inflammatory genes (CCL1, CCL2, CCL7, CXCL1, CXCL2, CXCL3, CXCL5, CXCL8, and NAMPT). Interestingly, NAMPT is involved in endothelial inflammation recruiting leukocyte and inducing vascular smooth muscle inflammation^17^.

GAPDH, a marker of T cell activation^18^, was significantly down-regulated after stimulation with both types of EVs, but PDGF-EVs decreased its expression significantly more than cEVs (RQ 0.3 vs. cEVs, p = 0.049). PDGF-EV also significantly down-regulated MIF expression that has been described to induce a pro-inflammatory response^19^.

PDGF-EVs significantly up-regulated CSF (RQ 2.3 vs. control PBMC, p = 0.009) and lncRNA MALAT1 (RQ 2.4 vs. control PBMC, p = 0.021) expression in PBMC.

**Figure 3:**
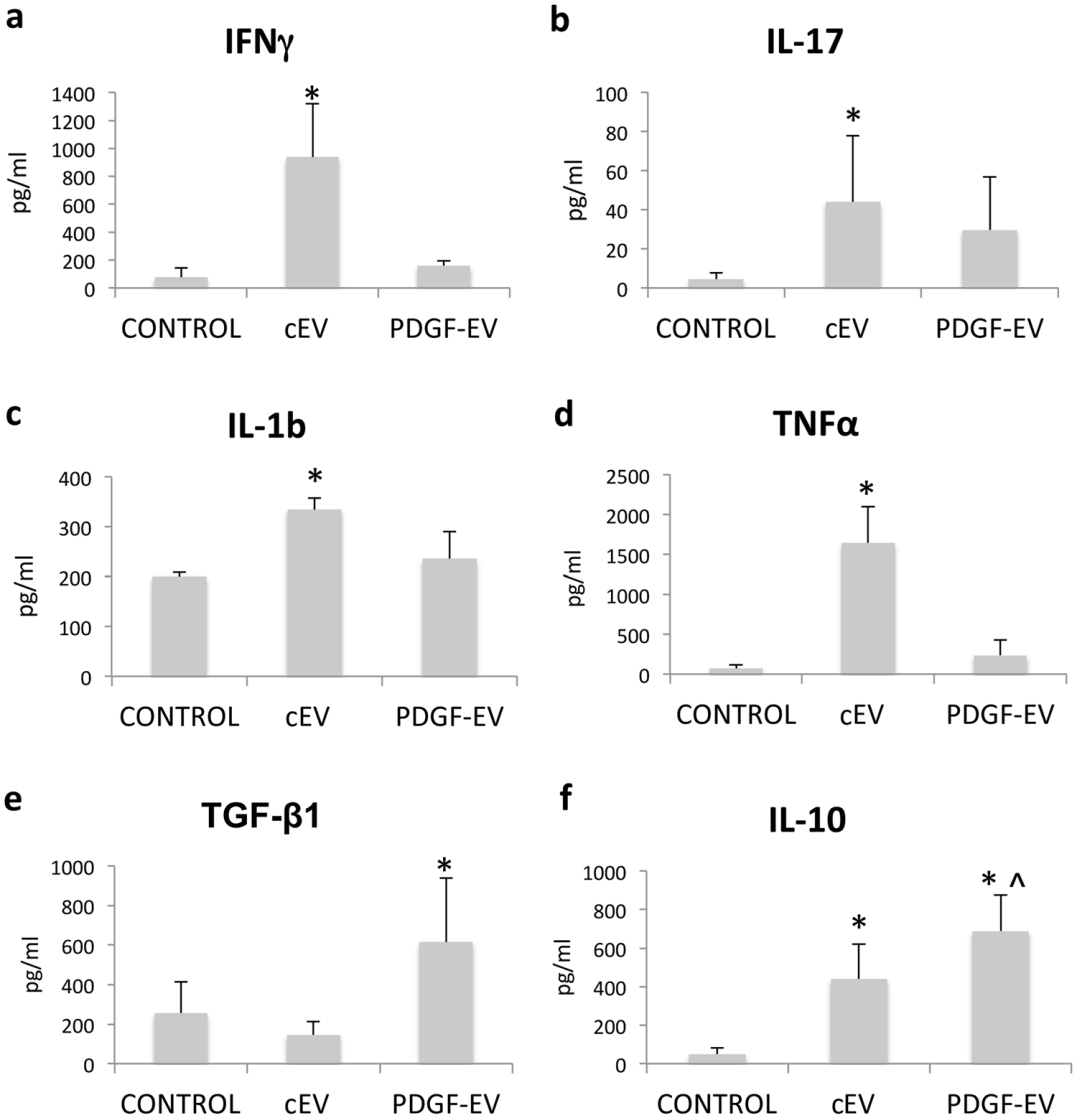
Cytokine secretion by PBMC stimulated with cEVs or PDGF-EVs. Concentration of pro-inflammatory factors IFNγ (**a**), IL-17 (**b**), IL-1β (**c**), and TNFα (**d**), as well as anti-inflammatory factors TGF-β1 (**e**) and IL-10 (**f**) was measured by ELISA in conditioned media of PBMC stimulated with EVs (mean ± SEM, *p < 0.05 vs. control PBMC, ^p < 0.05 vs. cEVs, n = 8).

The data from PCR arrays suggest that both types of EVs activated PBMC, but stimulation with PDGF-EVs could induce an anti-inflammatory phenotype. To confirm this hypothesis at the protein level, we performed the analysis of several cytokines by ELISA. This analysis revealed that cEVs significantly enhanced the production of pro-inflammatory factors IFNγ, IL-1b, IL-17, and TNFα by PBMC, whereas PDGF-EVs did not (Fig. 3a–d). Both types of EVs up-regulated the secretion of IL-10, but PDGF-EVs enhanced it significantly more (RQ 1.6 vs. cEVs, p = 0.002) relative to cEVs (Fig. 3f). PDGF-EVs also significantly increased TGF-β1 production by PBMC, whereas cEVs did not (Fig. 3e).

### PDGF-EVs decrease PBMC adhesion on endothelium and promote Treg formation

**Figure 4:**
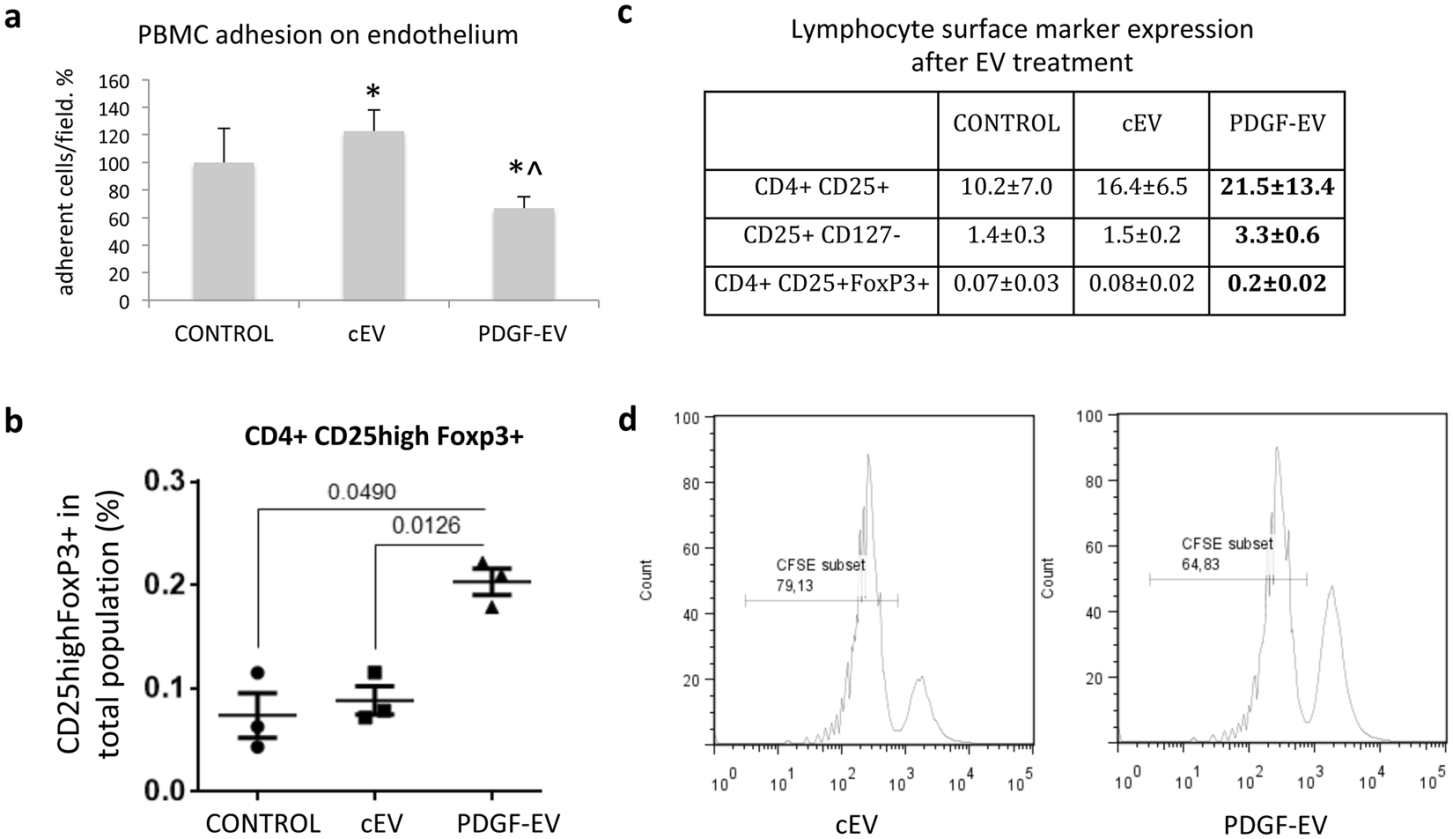
EV influence on PBMC. (**a**) PBMC adhesion on endothelium. PBMC and HUVEC were separately pre-stimulated with EVs 24 hours before, and then PBMC were labeled with PKH26GL red fluorescent cell linker and were added to HUVEC for 1 hour. After washing, adherent cells were counted by digital analysis (results mean ± SEM, *p < 0.05 vs. control PBMC, ^p < 0.05 vs. cEVs, n = 8). (**b**) The lymphocyte surface marker expression after EV treatment (mean % of total lymphocyte population ± SEM, in bold - p < 0.05 vs. control PBMC, n = 5). (**c**) The diagram of CD25high +/FoxP3 + expression on CD4 + cells (p-value between groups is indicated, n = 3). (**d**) The representative FACS image of CFSE labeled T cells after 5-day culture with Treg, isolated from PBMC stimulated by cEVs or PDGF-EVs, the percentage of proliferated cells is indicated.

As was mentioned before, cEVs induced the expression of NAMPT in PBMC, which is described as a factor implicated in leukocyte adhesion on endothelium. On the other hand, TGF-β1 and IL-10, up-regulated by PDGF-EVs, are described as factors able to reduce PBMC adhesion on endothelium^20–22^. To evaluate whether PDGF-EVs modulate the adhesion of labeled PBMC to the endothelium, we pre-incubated separately overnight PBMC and human umbilical vein endothelial cells (HUVEC) with ASC-EVs. PBMC and HUVEC were then co-incubated, and PBMC adhesion was evaluated. We found that cEVs significantly enhanced PBMC adhesion on HUVEC, whereas PDGF-EVs significantly decreased it (Fig. 4a).

Since PDGF-EVs enhanced secretion of TGF-β1 and IL-10, known to induce Treg differentiation and activation^23^, we investigated by FACS Treg differentiation. A significant increase of CD4^+^ CD25^+^ FoxP3^+^ and CD25^+^ CD127^−^ lymphocyte populations in PBMC treated with PDGF-EV relative to cEVs (Fig. 4b,c) was observed. We confirmed the functional activity of Treg by the test of activated T cells proliferation inhibition (Fig. 4d).

### PDGF-EVs protect muscles from acute ischemic injury and decrease inflammation *in vivo*

**Figure 5:**
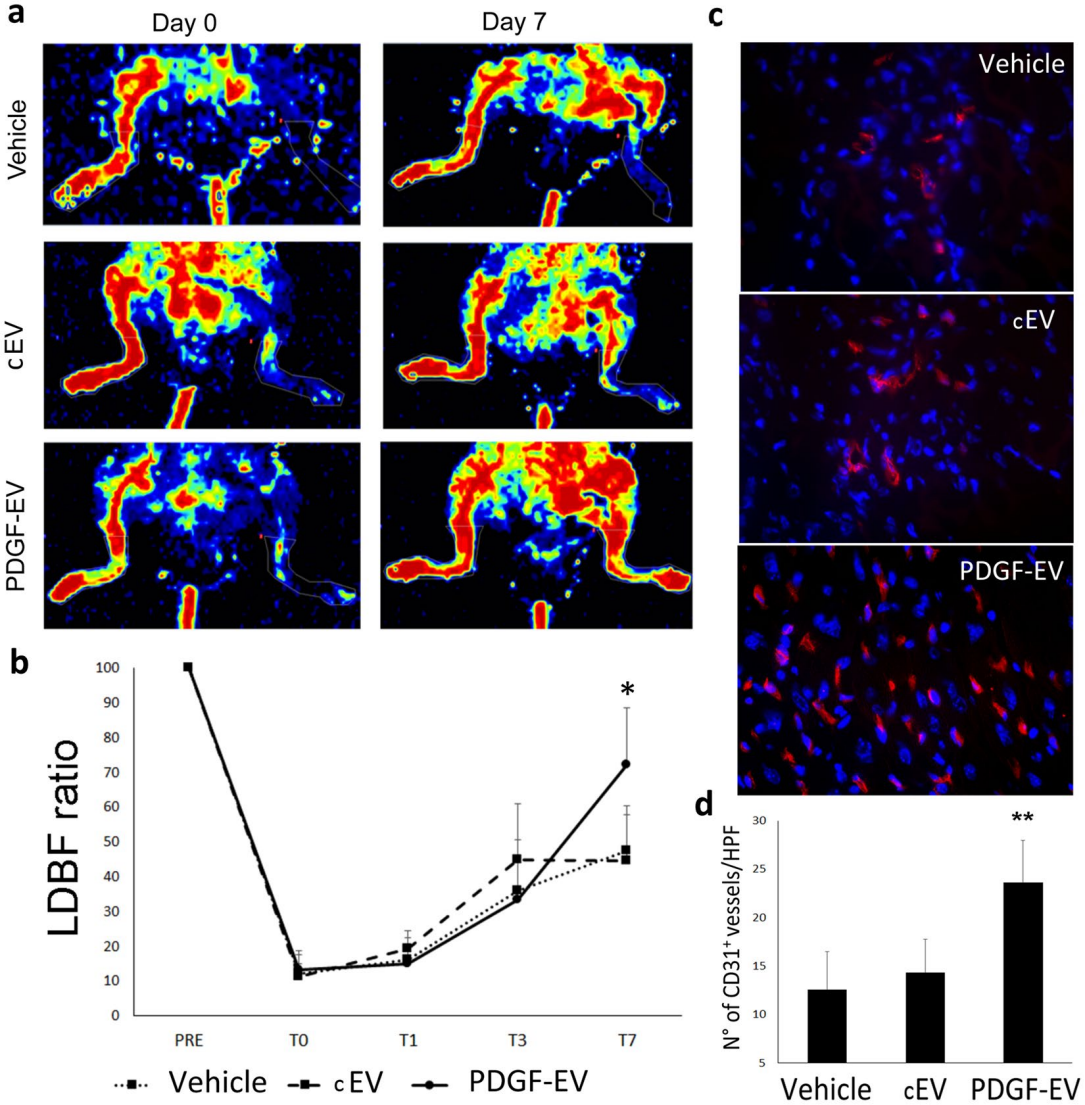
PDGF-EVs increase capillary density *in vivo* after acute hindlimb ischemia. (**a**) The representative images of LDBF obtained immediately after surgery (day 0) and 7 days after surgery (day 7) in control mice treated with PBS (vehicle) and in mice treated with cEVs or PDGF-EVs. (**b**) The quantitative analysis of blood perfusion measured by LDBF (mean ± SEM; *p < 0.05, n = 10). (**c**) The representative immunofluorescence images (confocal microscopy) of capillary density stained with anti-CD31 mouse antibodies, red fluorescence (Original Magnification: × 400). (**d**) The quantitative analysis of the capillary density of control ischemic tissue and EV-treated hindlimbs at day 7 after surgery indicates that PDGF-EVs significantly increase capillary density compared to vehicle or cEVs (mean ± SEM; **p < 0.01 vs vehicle, n = 10).

To investigate the role of PDGF-EVs *in vivo* we used a mouse model of acute hindlimb ischemia. As shown in Fig. 5, PDGF-EVs significantly enhanced blood perfusion detected by Laser Doppler relative to cEVs (Fig. 5a,b). Increased angiogenesis was confirmed by the staining of CD31^+^ cells within muscles.

**Figure 6:**
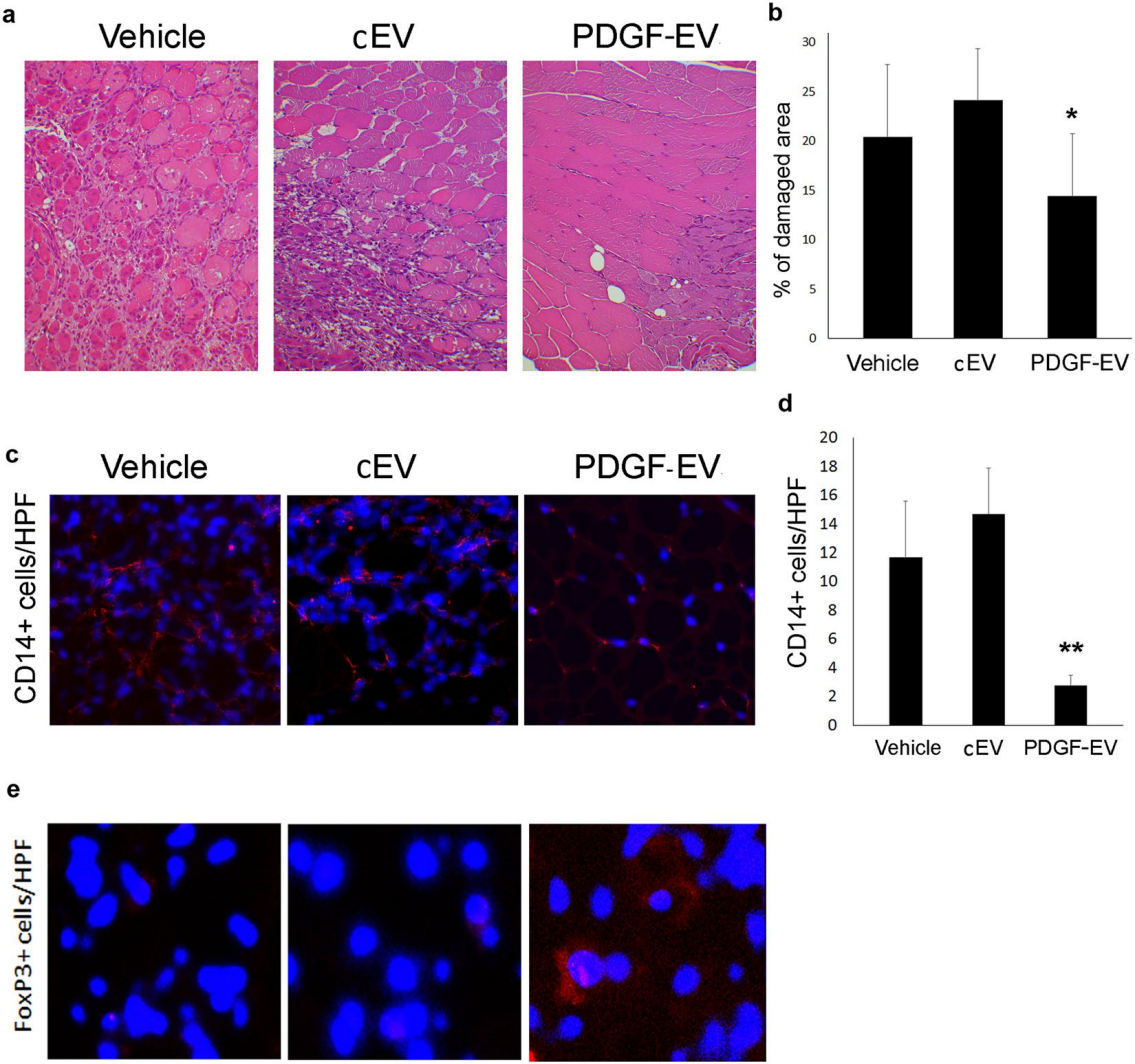
PDGF-EVs protect muscle tissue from acute injury *in vivo* and reduce inflammation. (**a**) The representative hematoxylin-eosin images of gastrocnemius muscles of an ischemic hindlimb of the vehicle, cEV, or PDGF-EV treated animals 7 days after surgery (Original Magnification: × 200). (**b**) The quantitative analysis of muscle damage areas in hematoxylin-eosin stained gastrocnemius muscles of control and EV-treated ischemic hindlimbs at day 7 after surgery. PDGF-EVs significantly reduce muscle damage compared to vehicle or cEVs (mean ± SEM; *p < 0.05 vs. vehicle). (**c**) The representative immunofluorescence images (confocal microscopy) of CD14 + cells infiltration (red fluorescence) in gastrocnemius muscles of ischemic hindlimbs. (**d**) The quantitative analysis of immune cell infiltration in ischemic muscle tissue of control and EV-treated animals (mean ± SEM; **p < 0.01 vs. Vehicle). (**e**) The representative immunofluorescence images (confocal microscopy) of FoxP3 + cells (red fluorescence) in gastrocnemius ischemic muscles of control and EV-treated animals.

The most relevant effect of PDGF-EVs was observed on muscle tissue protection from ischemia and on inhibition of monocyte infiltration. The histological analysis of gastrocnemius muscles 7 days after the induction of hindlimb ischemia revealed that the mice treated with vehicle or cEVs were characterized by diffuse necrotic area and by an intensive inflammatory cell infiltration (CD14^+^ cells) in the injured muscle tissue. In contrast, muscles of animals treated with PDGF-EVs were almost normal with regular centronucleated muscle fibers in more than 90% of the muscle cross-sections (Fig. 6a,b). Infiltration of CD14^+^ cells was significantly reduced in PDGF-EV treated animals (Fig. 6c,d). Moreover, only in muscles obtained from PDGF-EV treated mice were detected few Treg (Fig. 6e).

## Discussion

After tissue injury, the switch from inflammation to immunosuppression is linked to the enhanced concentration of PDGF^8^ and to the increased number and activity of Treg^24^. It was previously shown that PDGF could increase Treg formation through dendritic cells induction^25^. Mesenchymal stem cells and, in particular, ASC express the high level of PDGF receptors. Our data suggested that PDGF, known to regulate ASC pro-angiogenic and pro-regenerative properties, could also modulate the immunomodulatory characteristics of ASC.

Pro-inflammatory and anti-inflammatory properties of ASCs have been reported. ASCs from healthy subjects were reported to have anti-inflammatory pro-regenerative properties when administered in different disorders^26–30^. On the other hand, obesity, diabetes, and chronic inflammation, as well as culture conditions, may transform the phenotype of these cells from anti-inflammatory to pro-inflammatory^31,32^. Moreover, the pro-angiogenic potential of ASC-EVs was shown to be impaired by obesity^33^. Therefore, the environmental conditions may modulate the biological activity of both ASC and ASC-derived EVs^34^.

In the present study, we found that ASC derived from patients undergoing non-neoplastic elective surgery in basal culture conditions have a molecular profile characterized by the expression of both pro-inflammatory and anti-inflammatory molecules. This balance was changed by ASC stimulation with PDGF. In fact, PDGF induced enrichment in ASC-EVs of anti-inflammatory and immunomodulatory proteins such as TGF-β1 and IL-10. (Supplementary Table S1 and Fig. S3b).

The comparative analysis of miRNA pattern of cEVs and PDGF-EVs showed enrichment of miRNAs relevant to immunomodulation (TGF and TNF signalings) in PDGF-EVs, whereas cEVs contained more miRNAs regulating fatty acid metabolism. By comparison of miRNA patterns with the protein content of EVs, we found several potential reciprocal pairs miRNA-target proteins. For example, cEVs contain miR-1 and miR-299 and reduced expression of target VEGF and TIMP3. In contrast, PDGF-EVs that did not express miR-1 and miR-299 showed an increased expression of VEGF and TIMP3. On the other hand, cEV but not PDGF-EVs carried miRNAs that potentially target precursor of TGF-β LTBP1 (miR-1, miR-541-3p, and miR-659-3p), which was hyper-expressed in PDGF-EVs.

Both types of ASC-EVs, cEVs and PDGF-EVs, carried proteins, known to activate immune cells, such as TLRs, interleukins and their receptors, IFNγ, CSFs, PlGF, CRP, and others (see Supplementary Table S2).

In comparison with cEVs, PDGF-EVs could induce the secretion of anti-inflammatory factor TGF-β1 by PBMC and to decrease the secretion of pro-inflammatory factors IL-1b, IL-17, IFNγ, and TNFα. Moreover, we had noticed that cEVs significantly enhanced *in vitro* PBMC adhesion on endothelium, probably by increasing NAMPT expression^17^, whereas PDGF-EVs inhibited it.

Transient activation of immune cells and inflammation may be beneficial *in vivo* since may promote angiogenesis and tissue regeneration. However, an unbalance between the pro-inflammatory and anti-inflammatory molecules may be detrimental. Taken together, our results suggest that PBMC activation by ASC-EVs is a normal reaction, but PDGF stimulation shift the EV phenotype to a more anti-inflammatory phenotype.

Indeed, *in vivo* cEVs increased the infiltration of CD14^+^ cells in the injured tissue. However, since we sacrificed the animals on day 7, we may have missed a stronger initial inflammatory response. At variance of cEVs, PDGF-EVs significantly decreased the infiltration of CD14^+^ cells in injured tissue and induced the formation of Treg. Studies in the literature indicate that muscle tissue regeneration is linked to immune suppression and Treg functions^24,35^. Our data suggested that PDGF-EVs, which carried TGF-β1 and lncRNA MALAT1 and induced their expression in PBMC, could protect muscle tissue from ischemic injury since these factors are involved in Treg formation^36–41^. We also demonstrated that PDGF-EVs expressed significantly more TRAIL and TROY, which could induce apoptosis of activated CD8^+^ T cells and reduce immune response^42^. On the other hand, cEVs carried miR-1204, miR-1272, miR-1, and miR-330-5p that could target TGF-β1 according to miRwalk. We suggested that cEV attracted immune cells and activated them, thus inducing regenerative process in the injured tissue, whereas PDGF-EVs protected muscle tissue from ischemia restoring blood supply and limiting inflammation. In conclusion, our study demonstrated that secretion of ASC-derived EVs could be regulated by PDGF, changing both protein and RNA composition and their functions.

## Materials and Methods

### Isolation of ASC-EVs before and after PDGF stimulation

ASCs were isolated by enzyme digest as described previously^43^. Briefly, subcutaneous fat tissue from 6 patients submitted to non-neoplastic elective abdominal surgery (1 female and 5 males, mean age 51 ± 8) was washed in sterile PBS and was minced into pieces of 2 mm in diameter in a Petri dish. The minced tissues were transferred to a conical tube along with 220 U/mL collagenase I type (Worthington Biochemical, Lakewood, NJ), and 40 U/mL dispase (Invitrogen Corporation, Germany). The tissue was digested at 37 °C for 30 min with constant mixing. Enzyme activity was neutralized with DMEM, containing 10% fetal bovine serum (FBS, Lonza), 100 units/ml penicillin, 0.1 mg/ml streptomycin, and 0.25/ml μg amphotericin B (antibiotic/antimycotic solution, Sigma). The digested suspension was centrifuged at 900 g for 10 min to obtain a high-density stromal vascular fraction pellet. The cell pellet was resuspended in MSCBM complete medium (Lonza) and cultured at 37 °C in 5% CO_2_ incubator. After two days the medium was changed, and the cells were cultivated until 100% confluency (normally reached in 4–5 days). ASC characterization was performed by FACS analysis for the positive expression of mesenchymal markers (CD105, CD73, CD90), negative expression of hematopoietic markers (CD31) and by differentiation into adipogenic, osteogenic, and chondrogenic phenotypes as previously described^5,44^. For our experiments, we used cells after 2–8 passages.

For EV isolation ASCs were cultured in FBS-free DMEM for 18 hours. After washing, the cells were incubated in fresh FBS-free DMEM supplemented or not with PDGF (PDGFbb, Peprotech, 20 ng/ml) for additional 6 hours. Cells were then extensively washed to remove PDGF and fresh FBS-free DMEM was added, and cell-free conditioned medium for EV collection was obtained after 24-hour incubation by 300 g centrifugation 30 minutes at room temperature. To remove cell debris and apoptotic bodies cell-free supernatant was submitted to further centrifugation at 3000 g for 30 minutes at room temperature. Previously we showed that 10,000 g and 100,000 g fractions of EVs did not show a significant difference in the biological activity^4,45^ therefore we decided to use a total 100,000 g fraction for further experiments. Supernatants were submitted to ultracentrifugation twice for 2 hours at 100,000 g at 4 °C using the Beckman Coulter Optima L-100K Ultracentrifuge with the rotor type 45 Ti 45000RPM. The EV pellet was resolved in DMEM supplemented with 1% of DMSO, filtered with PES membrane filters (0.22 μm, Millipore) and then stored at −80 °C until further use. After thawed, EV aliquots were resuspended in PBS and analyzed using the Nanoparticle tracking analysis (NTA) by NanoSight NS300 system (Malvern Instruments, Ltd) to determine particle concentrations. NTA is a method of visualization and analysis of particles in liquids, by relating the rate of Brownian motion to particle size. Isolated particles were visualized by the light they scatter when illuminated by laser light. The rate of particle movement was linked to the size of the particles. Each sample was run in triplicate on different file recorded for 30 seconds and results were expressed as the mean of the three determinations. EVs were also analyzed by FACS (CytoFlex, Beckman Coulter), western blot and transmission electron microscopy.

### FACS analysis of EVs

A pool of approximately 5 × 10^10^ particles/100 μl was incubated with antibodies for 30 minutes at 4 °C. Then the final volume was increased to 500 μl and FACS analysis was performed at a low flow rate, with 10,000 events using CytoFlex from Beckman Coulter. The instrument was calibrated with Cytoflex Daily QC Fluorospheres according to the manufacturer’s instructions followed by Megamix-Plus FSC reagent (0.1, 0.3, 0.5, and 0.9 µm; Biocytex; Diagnostica Stago) and FlowCount beads. The used antibodies were specific for CD63 (FITC, BD, Pharmigen, #557288), CD81 (PE, BD Pharmigen, #555676), CD105 (FITC, Miltenyi, #130-098-774), ICAM (BD Pharmigen, #559771), VE-cadherin (FITC, BD Pharmigen, #560874), CD47 (FITC, BD Pharmigen,#556044), HLA class I (PE, BD Pharmigen, #560964), HLA class II (FITC, BD Pharmigen, #347400), Annexin V (FITC, Bender, #BMS306FI) was used for the characterization of EVs. Buffer alone with antibodies was analyzed prior to sample acquisition to ensure that any background noise was eliminated from the gates used for post-acquisition analysis. Post-acquisition analysis was carried out using CytExpert analysis software (Beckman Coulter). Representative plots showing EV populations are shown in Fig. 1g–I, and Supplementary Fig. S1c–h.

### ELISA assay

Relative quantification of CD63- and CD81- positive EVs was performed using the ELISA assay. Briefly, the suspensions of EVs, collected from an equal quantity of non-stimulated or PDGF-stimulated ASC, were incubated overnight at 4 °C on protamine sulfate coated 96 well plates (Cosmo Bio Co, LTD). According to NTA 1 × 10^7^ particles/100 μl was added per well. After washing with PBS, wells were stained with biotinylated antibodies against to CD63 (Miltenyi, #130-100-168) or CD81 (Miltenyi, #130-107-918).

### Protein array

For protein array, 400 ml of conditioned media from an equal amount of non-stimulated or PDGF-stimulated ASC was used. Purified cEVs and PDGF-EVs were lysed in an equal volume of 2x Cell Lysis Buffer (RayBiotech, Inc), and aliquots (1000 ng of protein measured by BCA Protein Assay Kit, Thermofisher) were used for the protein array according to manufacturer instructions for AAH-BGL-100004 Human L1000, Glass Slide (RayBiotech). The array provides detection of 1000 secreted proteins. We performed two independent experiments, comparing two different samples of cEVs and PDGF-EVs. Proteins, which RQ (PDGF-EV vs. cEVs) was higher than 2 in both experiments, were considered as consistently up-regulated (65 proteins). Proteins, which RQ was lower than 0.5 in both experiments, were considered as consistently down-regulated (15 proteins). Proteins consistently expressed with RQ between 0.5 and 2 were described as equally expressed proteins (228 proteins, Supplementary Table S1). Proteins that were detected up-regulated in one experiment and down-regulated in the other were not taken into consideration. Bioinformatic analysis of up- or down-regulated proteins was performed using Reactome (https://reactome.org).

### RNA extraction and cDNA synthesis

Total RNA from EV samples and PBMC was extracted using an RNAeasy kit (Qiagen), then RNA was quantified spectrophotometrically (Nanodrop ND-1000, Wilmington DE). Approximately 300 ng of RNA was reverse transcribed into complementary DNA (cDNA) using miScript II RT Kit (Qiagen) according to manufacturer protocol.

### MicroRNA array analysis

MicroRNA (miRNA) expression levels in control or PDGF-EVs were analyzed using the Applied Biosystems TaqMan® Array Human MicroRNA A/B Cards (Applied Biosystems, Foster City, CA) to profile 754 mature miRNAs by qRT-PCR. The kit used gene-specific stem-loop reverse transcription primers and TaqMan probes to detect mature miRNA transcripts in a 2-step real-time reverse-transcription PCR assay. Briefly, single-stranded cDNA was generated from total RNA sample (50 ng) by reverse transcription using a mixture of looped primers (Multiplex RT kit, Applied Biosystems) following the manufacturer’s protocol. The RT reactions were then diluted and mixed with a Taqman universal master Mix (Applied) in a ratio 1:1, loaded in the TaqMan microfluid card to complete the qRT-PCR experiments. All reactions were performed using the QuantStudio 12 K Flex real-time PCR instrument (Applied Biosystems) equipped with a 384 well reaction plate. Raw Ct values were calculated using the QuantStudio 12 K Flex software version 1.2.2 using automatic baseline and threshold. We have analyzed miRNA expression in 3 samples of cEVs and 3 samples of PDGF-EVs. Only miRNAs that were present in all cEV samples and/or in all PDGF-EV samples were considered as evidently expressed. All miRNAs that were amplified after 35 cycles of PCR were classified as non-expressed. Only miRNAs that were detected or not in all three replicates for cEVs and/or PDGF-EVs were taken into consideration (Supplemented Table 2).

Analysis of differential expression was performed using Expression Suite Software (ThermoFisher). For the further bioinformatics analysis, we selected miRNAs expressed exclusively in control or PDGF-EVs at a high level (Ct < 31) or significantly up-regulated in some of these types of EVs (in bold, Supplementary Table S2). Bioinformatic analysis was performed using the Diana tool mirPath V3 (http://snf-515788.vm.okeanos.grnet.gr) or miRWalk software (http://mirwalk.umm.uni-heidelberg.de). KEGG pathways and GO groups overrepresented by miRNAs from cEVs or PDGF-EVs are shown in Supplementary Table S3.

### Long non-coding RNA analysis of EVs

Profile of the lncRNA content of ASC-released EVs was carried out using the SBI’s LncProfilers qPCR kit. To enhance qPCR assay performance, the cDNA synthesis kit includes a step of polyadenylation of all the lncRNAs before cDNA conversion using the tagged oligo dT adaptor and random primers. This combined RNA tailing and oligo dT plus random primers boosts cDNA yield significantly and enables strand-specific lncRNA qPCR. A profile of 96 lncRNAs was performed using the QuantStudio 12 K Flex instrument. Three endogenous genes (GAPDH, Actin, and Laminin) and two RNU (RNU-43 and RNU-6b), used as controls, were present in each plate. Analysis of differential expression was performed using Expression Suite Software (ThermoFisher).

qRT-PCR was used to confirm some miRNAs screened by microarray analysis (Supplementary Fig. S3). Briefly, 200 ng of input RNA from all samples were reverse transcribed with the miScript Reverse Transcription Kit. cDNA was used to detect and quantify RNA of interest by qRT-PCR using the miScript SYBR Green PCR Kit (all from Qiagen, Valencia, CA, USA). All samples were run in triplicate using 3 ng of cDNA for each reaction as described by the manufacturer’s protocol (Qiagen). miRNA specific primers to miR-29a, miR-21-5p, miR-126-3p, miR-92a-3p, miR-145-5p, or lncRNA MALAT1 were used in separate reactions. Specific primers were provided from the primer bank (https://pga.mgh.harvard.edu/primerbank/). miRNA comparison between the two types of EVs was performed on the relative expression data normalized using the geometric mean value of 2 housekeeping genes (RNU6b and actin). Fold change in miRNA expression was calculated based on the normalized mean differences between different types of EVs (2−ΔCt). miRNAs with a raw Ct value greater than 35 were not included in the final data analysis.

### PBMC stimulation with ASC-EVs

The fresh PBMC were isolated by the density gradient centrifugation from heparinized blood samples obtained from eight healthy donors. PBMC were seeded in 6 well plates at a density of 10 × 10^6^/ml per well in serum-free AIM V medium. ASC-EVs were added to culture PBMC in the concentration of 1 × 10^10^/ml (approximately 1 × 10^3^ EV/cell) for 48 hours. As a control, we used PBMC stimulated with an equal amount of serum-free RPMI medium. After 48 hours the cultured PBMC and conditioned media were harvested for FACS, PCR and ELISA.

### EV internalization in PBMC

Control EVs or PDGF-EVs was labeled with PKH67GL green fluorescent cell linker (Sigma) dye for 30 min at 37 °C and then washed and ultracentrifuged at 100,000 g for 1 h at 4 °C. These labeled EVs were added to PBMCs (1 × 10^3^ EV/cell) for different times (10, 30 minutes, 1, 3 or 24 hours). The internalization of EVs into PBMCs was evaluated by FACS (CytoFlex, Beckman Coulter).

### Analysis of proliferation, gene expression and cytokine secretion by PBMC, stimulated with PDGF-EVs or cEVs

For proliferation test, FACS, and analysis of gene expression non-labeled EVs were used. The proliferation of PBMC was measured by cell counting and Cell Proliferation ELISA, BrdU (colorimetric) kit (Roche, 11647229001) according to manufacturer instructions.

PCR analysis of the inflammation genes, expressed by PBMC, was performed using the RT^2^ Profiler PCR Array (96 well format) for human inflammatory cytokines & receptors (Qiagen). ELISA of cytokines secreted by PBMC was performed using kits specific for TNFα, IL-10 TGF-β1, IFNγ, IL-17, IL-1b (DuoSet® ELISA Development Systems, R&D Systems) according to manufacturer instructions.

### Adhesion assay

Human umbilical vein endothelial cells (HUVEC) seeded in 24 well plate and grown till 100% confluence were stimulated with cEVs or PDGF-EVs in the concentration 1 × 10^8^/ml overnight. Fresh PBMC were cultured 24 hours with or without cEVs or PDGF-EVs in AIM V serum-free medium. PBMC were then labeled with PKH26GL red fluorescent cell linker (Sigma) according to manufacturer instructions. Labeled 160 × 10^3^ PBMCs in 0.5 ml of AIM V culture medium were added per well of confluent stimulated in the same manner HUVEC for 1 h at 37 °C. Non-adherent cells were removed by supernatant aspiration and two additional washing steps. Adherent cells were counted by digital analysis (Leica application suit V4.9). Four independent experiments were performed in duplicates; results were normalized in respect to non-stimulated PBMC and HUVEC and are expressed as mean % of cells counted in ten inverted microscope fields.

### PBMC FACS analysis

For the surface marker proteins antibodies against CD11b (FITC, BD Pharmigen), CD80 (PE, BD Pharmigen, #557227), CD127 (PE, BD Pharmigen, #557938), PDL1 (PE, BD Pharmigen, #557924) were used.

Treg were detected by FACS using monoclonal antibodies against human CD4 (PerCP/Cy5.5, BioLegend, #317428), CD25 (PE, BioLegend, #302606), and FoxP3 (FITC, eBioscience, #11-4777-42).

### A murine model of hindlimb ischemia and blood flow monitoring

C57 mice (C57BL/6 N from Charles River Laboratories), aged 11 to 13 weeks, were intramuscular anesthetized. Hindlimb ischemia was performed as previously described^46^. Briefly, the proximal end of the left femoral artery and the distal portion of the saphenous artery were ligated and dissected free and excised. Control or PDGF-EVs (10 × 10^6^) were intravenously injected immediately after ischemia followed by two intramuscular injections of EVs (5 × 10^6^) on day 1 and 2. Shortly after the surgery and 7 days after, blood perfusion was evaluated by Laser Doppler (LDBF analyzer, PeriScan PIM 3 System, Perimed) and animals were sacrificed for histological analysis. Tissue slices were stained with hematoxylin and eosin. Muscle samples were embedded in OCT compound (Bio-Optica), and capillary density and inflammatory cells were quantified using antibodies against CD31 (Abcam), CD14 (BD Pharmingen), and FoxP3 (Abcam). Alexa Fluor Texas Red (Molecular Probe) was used as secondary antibody.

### Statistical analysis

Statistical analysis was performed using SigmaPlot 11.0 Software. Differences between the treatment and control groups were analyzed using the paired and unpaired two-tailed Student’s t-tests when the distribution was normal. Data are expressed as mean ± SEM. We considered differences to be significant when p < 0.05.

### Ethics approval and consent to participate

Ethics approval for human ASC isolation was obtained from the Ethics Committee of A.O.U. Città della Salute e della Scienza di Torino, Turin, Italy (CS/100 – Protocol number 12175, February 4, 2014). Informed consent was obtained from all patients providing adipose tissue according to the Helsinki Declaration. The laboratory researchers had no direct contact with the participants in the present study (patients’ anonymity was guaranteed).

Animal studies and experimental protocols were conducted in accordance with the Italian National Institute of Health Guide for the Care and Use of Laboratory Animals (protocol no: 490/2105-PR). Mice were housed according with the Federation of European Laboratory Animal Science Association Guidelines and the Ethical Committee of the University of Turin. All experiments were approved by the “Animal Use and Care Committee” of the Turin University.

## Electronic supplementary material

Supplementary dataset

## Data Availability

All data are available within the manuscript or in the supplementary material.
