# Supplementary material for: PDGF enhances the protective effect of adipose stem cell-derived extracellular vesicles in a model of acute hindlimb ischemia
Source: Sci Rep. 2018 Dec 4;8:17458. doi: 10.1038/s41598-018-36143-3 (PMC6279818; doi:10.1038/s41598-018-36143-3)
Supplement: Supplementary file 1 — Supplementary dataset [file 41598_2018_36143_MOESM1_ESM.pdf]

PDGF enhances the protective effect of adipose stem cell-derived extracellular vesicles in a model of acute hindlimb ischemia

Tatiana Lopatina[1], tatiana.lopatina@unito.it
Enrica Favaro[1], enrica.favaro@unito.it
Cristina Grange[1], cristina.grange@unito.it
Massimo Cedrino[2], massimo.cedrino@unito.it
Andrea Ranghino[1], andrea.ranghino@unito.it
Sergio Occhipinti[1], sergio.occhipinti@unito.it
Sofia Fallo[1], sofia.fallo@unito.it
Fabrizio Buffolo[1], fabrizio.buffolo@gmail.com
Daria Gaykalova[4], dgaykal@jhmi.edu
Maria M Zanone[1], mariamaddalena.zanone@unito.it
Renato Romagnoli[3], renato.romagnoli@unito.it
Giovanni Camussi[1,2], giovanni.camussi@unito.it

[1] Department of Medical Sciences, University of Turin, Turin, Italy.

[2] 2i3T, Società per La Gestione Dell'incubatore Di Imprese e Per Il Trasferimento Tecnologico Dell'Università degli Studi di Torino, Scarl., Molecular Biotechnology Center (MBC), Turin, Italy

[3] General Surgery 2U, Liver Transplantation Center, AOU Città della Salute e della Scienza di Torino, University of Turin, Turin, Italy.

[4] Otolaryngology - Head and Neck Surgery, The Johns Hopkins University School of Medicine. 1550 Orleans street, Rm 5M06, CRBII, Baltimore, MD 21231

Supplementary figure S1. Characterization of cEVs and PDGF-EVs.

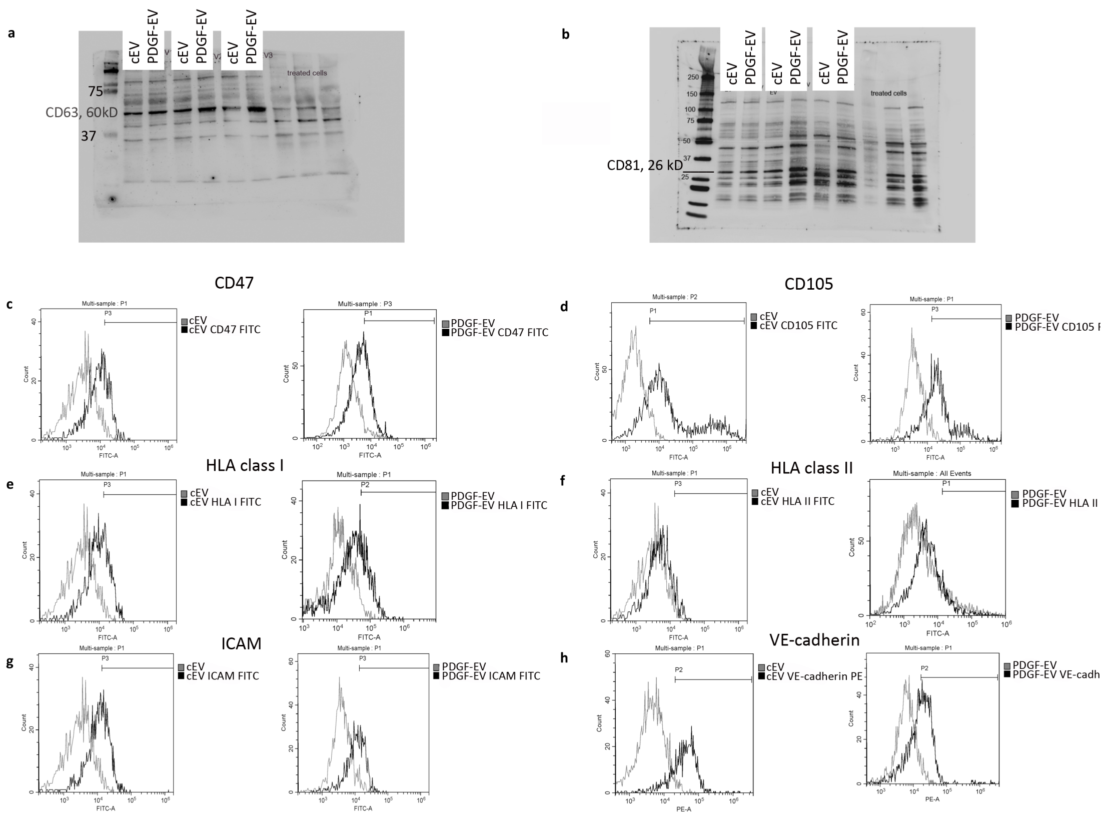

Supplementary figure S1. Characterization of cEVs and PDGF-EVs. (a, b) representative raw images of western blot analysis confirmed the expression of CD63 (a) and CD81 (b) on EVs, 10μg of EV protein per line; (c-h) representative images of FACS analysis of CD47 (c), CD105 (d), HLA class I (e), HLA class II (f), ICAM (g), VE-cadherin (h) expression on cEVs (left) and PDGF-EVs (right). Grey line – EVs, incubated with appropriated IgG, black line – EVs stained with appropriated fluorochrome-conjugated antibodies.

Supplementary table 1. Relative enrichment of proteins in cEVs and PDGF-EVs.

PROTEIN		PDGF-EV (1)	cEV (1)	RQ (1)	PDGF-EV (2)	cEV (2)	RQ (2)	MEAN RQ
**VEGF**	NM_001025366	16778	1384	12.12	344	254	1.36	6.7
**NAP-2**	NM_002704	6183	531	11.65	63	55	1.13	6.4
**TRAIL / TNFSF10**	NM_003810	1977	353	5.60	47	12	3.77	4.7
**VEGF-D**	NM_004469	1518	189	8.01	69	68	1.01	4.5
**XEDAR**	NM_021783	11454	1588	7.21	885	803	1.10	4.2
**TROY / TNFRSF19**	NM_018647	857	237	3.61	70	16	4.30	4.0
**TLR4**	NM_138554	431	115	3.73	51	14	3.60	3.7
**CCR7**	NM_001838	270	64	4.20	44	14	3.11	3.7
**I-TAC / CXCL11**	NM_005409	18080	3566	5.07	189	88	2.15	3.6
**S100A10**	NM_002966	1436	269	5.35	63	37	1.70	3.5
**TGF-beta RIII**	NM_003243	816	234	3.48	25	8	3.25	3.4
**RANTES**	NM_002985	1289	243	5.30	48	39	1.23	3.3
**CK-MB**	NM_001823	909	167	5.40	141	127	1.11	3.3

**VEGF-C**	NM_005429	5105	931	5.48	297	296	1.00	3.2
**VEGF R2 (KDR)**	NM_002253	1544	306	5.04	59	46	1.29	3.2
**CD30 Ligand / TNFSF8**	NM_001244	12240	2670	4.58	105	66	1.60	3.1
**C3a**	NM_000064	2229	494	4.50	284	184	1.54	3.0
**IL-23**	NM_016584	2570	835	3.08	158	54	2.92	3.0
**Siglec-5/CD170**	NM_003830	2624	557	4.71	187	177	1.05	2.9
**PDGF-D**	NM_025208	1642	363	4.53	37	34	1.09	2.8
**TREM-1**	NM_018643	2422	571	4.24	158	115	1.38	2.8
**TIMP-3**	NM_000362	4416	1001	4.41	260	229	1.13	2.8
**NCAM-1 / CD56**	NM_181351	1502	377	3.98	64	42	1.54	2.8
**NM23-H1/H2**	NM_000269	323	76	4.30	48	42	1.13	2.7
**Orexin A**	NM_001525	2430	602	4.03	49	37	1.32	2.7
**TL1A / TNFSF15**	NM_005118	1460	354	4.12	41	37	1.12	2.6
**TGFb1**	NM_000660	66935	1632	4.10	4815	4532	1.06	2.5
**MICA**	NM_001177519	1291	354	3.65	72	50	1.45	2.5
**Insulysin / IDE**	NM_004969	1341	348	3.86	65	57	1.15	2.5
**Thrombopoietin (TPO)**	NM_000460	1001	275	3.63	59	44	1.35	2.5
**Neurturin**	NM_004558	846	272	3.11	68	37	1.85	2.5
**sFRP-1**	NM_003012	1035	338	3.06	26	14	1.89	2.5
**TRANCE**	NM_033012	1458	378	3.85	105	102	1.03	2.4
**MDC**	NM_002990	1518	403	3.77	41	38	1.07	2.4
**MMP-10**	NM_002425	1556	414	3.76	92	89	1.04	2.4
**TLR2**	NM_003264	1406	408	3.45	36	27	1.34	2.4
**MMP-9**	NM_004994	3565	976	3.65	206	187	1.10	2.4
**NeuroD1**	NM_002500	1163	314	3.71	41	40	1.02	2.4
**I-309**	NM_002981	1160	379	3.06	60	36	1.65	2.4
**IL-21 R**	NM_021798	980	309	3.17	38	25	1.52	2.3
**Ubiquitin+1**	NM_018955	34834	9751	3.57	3785	3422	1.11	2.3
**Factor XIII B**	NM_001994	637	240	2.70	101	57	1.76	2.2
**Lymphotoxin beta / TNFSF3**	NM_009588	715	254	2.82	63	39	1.61	2.2
**LDL R**	NM_000527	2626	790	3.30	149	146	1.02	2.2
**IL-13 R alpha 2**	NM_000640	802	321	2.50	53	30	1.80	2.1
**Cytokeratin 19**	NM_002276	3935	2286	1.70	5141	1986	2.59	2.1
**MMP-7**	NM_002423	2463	840	2.93	141	105	1.35	2.1
**Progranulin**	NM_002087	3801	1216	3.12	115	104	1.11	2.1
**MMP-14**	NM_004995	1967	625	3.15	335	310	1.08	2.1
**CD71**	NM_001128148	15834	6821	2.30	2998	1579	1.90	2.1
**Cystatin A**	NM_005213	985	317	3.10	77	70	1.10	2.1
**APC**	NM_000038	847	381	2.20	612	311	1.96	2.1
**Kremen-2**	NM_024507	2631	842	3.12	154	153	1.00	2.1
**IP-10**	NM_001565	1088	362	3.01	109	98	1.10	2.1
**Cathepsin D**	NM_001909	802	257	3.10	71	71	1.01	2.1
**BMP-9**	NM_016204	1670	566	3.00	177	165	1.07	2.0
**CD74**	NM_004355	860	319	2.70	126	92	1.37	2.0
**CD38**	NM_001775	973	444	2.20	152	82	1.86	2.0
**IL-19**	NM_153758	11943	4143	2.88	187	166	1.13	2.0
**E-Cadherin**	NM_004360	497	169	2.90	83	75	1.11	2.0

**BDNF**	NM_170735	1811	763	2.37	167	107	1.56	2.0
**Calsyntenin-1**	NM_001009566	737	262	2.80	84	74	1.13	2.0
**MIF**	NM_002415	2365	858	2.76	102	88	1.16	2.0
**Ceruloplasmin**	NM_000096	1606	845	1.90	423	211	2.00	2.0
IL-24	NM_006850	922	356	2.59	61	47	1.30	1.9
OX40 Ligand / TNFSF4	NM_003326	1013	358	2.83	97	95	1.02	1.9
IL-23 R	NM_144701	1470	539	2.73	92	82	1.12	1.9
LRG1	NM_052972	11893	4248	2.80	919	877	1.05	1.9
IL-1 R9	NM_017416	928	383	2.42	56	39	1.42	1.9
gamma-Thrombin	NM_000506	133	49	2.70	98	86	1.13	1.9
IGFBP-5	NM_000599	1008	371	2.70	82	73	1.12	1.9
MIP-1a	NM_002983	1211	446	2.71	101	91	1.10	1.9
Nanog	NM_024865	785	296	2.70	70	64	1.10	1.9
ADAMTS-18	NM_199355	216	81	2.70	86	79	1.09	1.9
DCBLD2	NM_080927	906	375	2.40	84	60	1.38	1.9
IL-13 R alpha 1	NM_001560	2342	886	2.64	362	334	1.09	1.9
BNIP2	NM_004330	1897	887	2.10	636	400	1.59	1.8
MMP-11 /Stromelysin-3	NM_005940	3585	1431	2.51	427	364	1.17	1.8
CFHR2	NM_005666	711	295	2.40	79	62	1.28	1.8
GMNN	NM_015895	5478	2583	2.10	806	517	1.56	1.8
ApoE3	NM_000041	844	352	2.40	99	79	1.25	1.8
Aldolase B	NM_000035	683	268	2.50	91	79	1.15	1.8
ApoM	NM_019101	826	331	2.50	82	71	1.14	1.8
LIMPII	NM_001204255	998	387	2.60	95	92	1.03	1.8
BMPR-IB / ALK-6	NM_001203	248	183	1.35	88	39	2.28	1.8
LBP	NM_004139	1052	468	2.25	69	50	1.37	1.8
MMP-13	NM_002427	1530	587	2.61	74	73	1.01	1.8
IL-7	NM_000880	17003	7546	2.25	5362	3970	1.35	1.8
Pro-Cathepsin B	NM_147780	1185	466	2.50	69	62	1.10	1.8
Btk	NM_000061	1508	792	1.90	541	318	1.70	1.8
IL-7 R alpha	NM_002185	1075	502	2.14	78	54	1.45	1.8
BMX	NM_001712	1393	639	2.20	284	205	1.39	1.8
CCR5	NM_000579	921	386	2.39	92	77	1.20	1.8
Erythropoietin R	NM_000121	735	292	2.50	75	70	1.08	1.8
PDGF R alpha	NM_006206	15002	6054	2.48	198	180	1.10	1.8
Aldolase C	NM_005165	2326	1122	2.10	405	277	1.46	1.8
CPN2	NM_000129	2839	1466	1.90	1065	641	1.66	1.8
CA 15-3	NM_002456	579	488	1.20	926	392	2.36	1.8
DMP-1	NM_001079911	408	162	2.50	56	54	1.04	1.8
Glypican 5	NM_004466	17063	9823	1.74	1732	964	1.80	1.8
Serpin I1	NM_001122752	743	346	2.10	47	33	1.43	1.8
CD36	NM_001001547	260	111	2.30	235	192	1.23	1.8
APN	NM_001150	984	400	2.50	154	151	1.02	1.8
EphA8	NM_020526	1219	480	2.50	205	202	1.01	1.8
Alpha Lactalbumin	NM_002289	1300	849	1.50	590	294	2.01	1.8
Calreticulin	NM_004343	988	413	2.40	113	102	1.10	1.8
FGF-21	NM_019113	659	360	1.83	61	37	1.67	1.8

ADAMTS-1	NM_006988	1037	574	1.80	231	136	1.70	1.8
PYY	NM_004160	1187	520	2.30	81	67	1.20	1.7
Caspase-3	NM_004346	639	268	2.40	78	72	1.09	1.7
S100 A8/A9	NM_002964. NM_002965	2910	1187	2.45	384	373	1.03	1.7
COCO	NM_152654	849	351	2.40	78	74	1.06	1.7
CA 125	NM_024690	1049	761	1.40	1254	614	2.04	1.7
TRPM7	NM_017672	496	212	2.30	49	43	1.14	1.7
EpCAM	NM_002354	659	280	2.40	80	77	1.04	1.7
ESAM	NM_138961	865	372	2.30	122	107	1.13	1.7
Endothelin Receptor A	NM_001166055	810	386	2.10	245	185	1.33	1.7
Galectin-3	NM_002306	1340	570	2.35	227	214	1.06	1.7
GPX3	NM_002084	857	352	2.40	74	74	1.00	1.7
IL-28A	NM_172138	987	444	2.23	97	82	1.17	1.7
MMP-24 / MT5-MMP	NM_006690	1555	692	2.25	453	397	1.14	1.7
CRTAM	NM_019604	879	379	2.30	207	194	1.07	1.7
IL-29	NM_172140	935	423	2.21	73	63	1.15	1.7
Kallikrein 11	NM_006853	771	335	2.30	99	96	1.02	1.7
Neuropeptide Y	NM_000905	481	206	2.30	58	57	1.02	1.7
Annexin A7	NM_001156	957	425	2.30	82	81	1.01	1.7
HB-EGF	NM_001945	962	521	1.84	73	50	1.47	1.7
CHI3L1	NM_001276	4053	2020	2.00	1859	1474	1.26	1.6
ADAMTS-19	NM_133638	755	456	1.70	508	328	1.55	1.6
CD44	NM_000610	613	294	2.10	198	175	1.13	1.6
GPX1	NM_000581	831	372	2.20	97	94	1.03	1.6
sFRP-3	NM_001463	825	396	2.08	50	44	1.14	1.6
Hepcidin	NM_021175	1047	468	2.20	98	96	1.01	1.6
C5/C5a	NM_001735	1579	764	2.10	146	135	1.09	1.6
ADAMTS-4	NM_005099	640	298	2.10	169	156	1.08	1.6
ICAM-2	NM_000873	1009	484	2.09	55	50	1.09	1.6
S100A6	NM_002964	3154	1687	1.90	722	573	1.26	1.6
INSL3	NM_014215	1162	547	2.10	388	384	1.01	1.6
p21	NM_078467	2852	1494	1.90	729	613	1.19	1.5
IL-27	NM_145659	1624	796	2.04	116	113	1.03	1.5
IGF-I		1039	515	2.02	91	87	1.05	1.5
IL-2 R alpha	NM_000417	2277	1723	1.32	199	114	1.74	1.5
MCP-4 / CCL13	NM_005408	384	195	1.97	112	104	1.07	1.5
HSP27	NM_001540	917	448	2.00	150	145	1.04	1.5
Layilin	NM_178834	1550	789	2.00	168	166	1.01	1.5
Cathepsin B	NM_147780	3197	1592	2.00	142	140	1.01	1.5
EphA6	NM_001080448	3312	1771	1.90	387	352	1.10	1.5
IL-17C	NM_013278	1894	981	1.93	98	92	1.07	1.5
IL-17B R	NM_172234	1050	544	1.93	186	177	1.05	1.5
IGFBP-rp1 / IGFBP-7	NM_001553	4473	2397	1.87	181	163	1.11	1.5
EphB4	NM_004444	567	321	1.80	78	68	1.14	1.5
ADAMTS-L2	NM_014244	284	155	1.80	119	105	1.14	1.5
FER	NM_005246	1821	975	1.90	249	243	1.03	1.5
EGF R / ErbB1	NM_005228	2831	1747	1.62	165	127	1.30	1.5

CRP	NM_000567	1382	907	1.50	634	447	1.42	1.5
Integrin alpha V	NM_002210	1699	924	1.80	1166	1053	1.11	1.5
IL-9	NM_000590	3684	2273	1.62	504	395	1.28	1.4
Tyk2	NM_003331	340	191	1.80	37	34	1.10	1.4
FLRG	NM_005860	571	365	1.56	60	45	1.33	1.4
6Ckine	NM_002989	781	516	1.51	136	98	1.38	1.4
GPBB	NM_002862	1295	796	1.60	407	325	1.25	1.4
ApoA4	NM_000482	1499	1055	1.40	567	390	1.45	1.4
Furin	NM_002569	127	77	1.60	131	106	1.24	1.4
11b-HSD1	NM_005525	500	285	1.80	183	180	1.01	1.4
CNTF	NM_000614	11569	8121	1.42	1018	748	1.36	1.4
IL-17	NM_002190	3687	2290	1.61	679	581	1.17	1.4
C2	NM_000063	2686	1709	1.60	828	707	1.17	1.4
C-peptide	NM_000207	1877	1630	1.20	1138	746	1.52	1.4
Endorphin Beta	NM_000939	746	460	1.60	287	258	1.11	1.4
Fetuin B	NM_014375	121	84	1.40	96	74	1.31	1.4
Fyn	NM_002037	2165	1311	1.70	314	312	1.00	1.4
ACK1	NM_005781	3091	1903	1.60	393	359	1.10	1.3
IFN-beta	NM_002176	1344	912	1.47	124	101	1.22	1.3
SOST	NM_025237	680	413	1.60	48	44	1.09	1.3
HCC-4 / CCL16	NM_004590	960	628	1.53	107	94	1.14	1.3
EG-VEGF / PK1	NM_032414	1107	1102	1.00	91	55	1.66	1.3
IL-1 R8	NM_014271	4196	2576	1.63	958	935	1.03	1.3
Angiopoietin-4	NM_015985	317	275	1.15	100	68	1.47	1.3
MCP-2	NM_005623	809	529	1.53	128	121	1.06	1.3
IGFBP-1	NM_001013029	916	585	1.57	38	38	1.02	1.3
HCR / CRAM-A/B	NM_019052	2101	1489	1.41	398	344	1.16	1.3
GRO	NM_001511.<br>NM_0020893.<br>NM_0020902.	32778	21903	1.50	9507	9128	1.04	1.3
Beta Defensin 4	NM_004942	365	280	1.30	176	144	1.22	1.3
BCMA / TNFRSF17	NM_001192	704	641	1.10	114	80	1.42	1.3
A1BG	NM_1307863	2265	1723	1.30	557	495	1.13	1.2
Eotaxin-3 / CCL26	NM_006072	626	449	1.39	39	38	1.03	1.2
ErbB2	NM_001005862	526	399	1.32	80	72	1.10	1.2
Alpha 1 AG	NM_000607	1310	922	1.40	472	464	1.02	1.2
Dkk-1	NM_012242	1919	1669	1.15	174	137	1.27	1.2
Insulin R	NM_000208	4002	3572	1.12	5434	4272	1.27	1.2
IL-1 alpha	NM_000575	21393	18520	1.16	3673	2995	1.23	1.2
FGF-20	NM_019851	1415	1072	1.32	161	154	1.05	1.2
SOX2	NM_003106	190	148	1.30	26	26	1.02	1.2
Calbindin	NM_004929	1351	1039	1.30	488	485	1.01	1.2
Creatinine	NM_001994	1809	1375	1.30	647	646	1.00	1.2
GLO-1	NM_0067082	759	602	1.26	75	73	1.03	1.1
IL-1 F10 / IL-1HY2	NM_173161	1798	1478	1.22	262	245	1.07	1.1
ApoC1	NM_000384	220	203	1.10	229	196	1.16	1.1
IL-8	NM_000584	26309	21327	1.23	11585	11436	1.01	1.1
EDG-1	NM_001400	6307	5809	1.09	1015	885	1.15	1.1
Alpha 1	NM_001633	87	70	1.20	93	91	1.02	1.1

Microglobulin								
Glut1	NM_006516	7683	7109	1.08	686	622	1.10	1.1
IL-21	NM_021803	21043	20359	1.03	11419	10452	1.09	1.1
ABL1	NM_007313	547	501	1.10	212	210	1.01	1.1
Glut5	NM_003039	8160	8383	0.97	1073	1113	0.96	1.0
FGF-8	NM_006119/ NM_033165	854	912	0.94	94	106	0.89	0.9
Flt-3 Ligand	NM_004119	1396	1460	0.96	205	242	0.85	0.9
HVEM / TNFRSF14	NM_003820	483	498	0.97	151	183	0.82	0.9
Activin B	NM_002193	166	209	0.79	103	104	0.99	0.9
CD14	NM_0005913	936	1203	0.78	115	118	0.98	0.9
Hepassocin	NM_004467	1456	1469	0.99	349	458	0.76	0.9
FGF Basic	NM_0020065	460	547	0.84	107	120	0.89	0.9
FGF-6	NM_020996	865	872	0.99	103	139	0.74	0.9
Decorin	NM_133507	995	1097	0.91	117	141	0.83	0.9
MMP-20	NM_004771	372	379	0.98	257	342	0.75	0.9
Granzyme A	NM_006144	40611	55760	0.73	21822	22021	0.99	0.9
IFN-gamma	NM_000619	37449	46979	0.80	15751	17140	0.92	0.9
CXCR6	NM_006564	674	738	0.91	152	191	0.80	0.9
FGF-12	NM_021032	1031	1072	0.96	153	206	0.74	0.9
Frizzled-1	NM_003505	1624	1975	0.82	283	322	0.88	0.9
Glut3	NM_006931	1148	1215	0.94	159	209	0.76	0.9
IL-2 R gamma	NM_000206	4724	4771	0.99	367	516	0.71	0.9
GDF11	NM_005811	8276	11391	0.73	9722	10011	0.97	0.8
ASPH	NM_004318	253	284	0.90	239	303	0.79	0.8
CD27 / TNFRSF7	NM_001242	662	772	0.86	79	96	0.83	0.8
GM-CSF R alpha	NM_172247	1094	1516	0.72	386	404	0.96	0.8
ErbB4	NM_005235	326	387	0.84	31	37	0.83	0.8
FAM3B	NM_058186	857	1069	0.80	181	210	0.86	0.8
AMICA	NM_153206	135	165	0.80	78	92	0.85	0.8
CXCL16	NM_022059	212	311	0.68	35	37	0.97	0.8
ENA-78	NM_002994	443	685	0.65	62	64	0.96	0.8
CCR4	NM_005508	541	631	0.86	168	226	0.74	0.8
FGF-BP	NM_005130	138	208	0.66	101	109	0.93	0.8
BMP-4	NM_130850	580	841	0.69	119	134	0.89	0.8
Follistatin	NM_013409	3079	3666	0.84	487	660	0.74	0.8
CEA	NM_006890	110	175	0.60	304	313	0.97	0.8
2B4	NM_016382	128	187	0.70	71	83	0.86	0.8
MTUS1	XM_372031	111	120	0.90	107	162	0.66	0.8
GDF3	NM_020634	20485	29318	0.70	3786	4434	0.85	0.8
CD200	NM_001004196	180	210	0.90	97	151	0.64	0.8
EDA-A2	NM_001399	13932	16770	0.83	2400	3397	0.71	0.8
GCSF	NM_172220	21008	28670	0.73	1867	2329	0.80	0.8
AR (Amphiregulin)	NM_001657	282	419	0.67	40	47	0.86	0.8
BTC	NM_001729	1969	3572	0.55	291	297	0.98	0.8
Cryptic	NM_032545	1752	2854	0.61	303	335	0.91	0.8
BMP-3	NM_001201	368	632	0.58	60	64	0.94	0.8
Frizzled-3	NM_017412	796	933	0.85	184	277	0.66	0.8

CCR9	NM_006641	287	308	0.93	80	140	0.57	0.8
CD30 / TNFRSF8	NM_001243	7699	10512	0.73	594	781	0.76	0.7
Cerberus 1	NM_005454	206	319	0.65	60	71	0.84	0.7
Amylin	NM_000415	1501	1971	0.80	248	379	0.65	0.7
CNDP1	NM_032649	79	96	0.80	181	277	0.65	0.7
FGF-17	NM_003867	2240	2887	0.78	407	605	0.67	0.7
CTLA-4 /CD152	NM_005214	1171	2120	0.55	335	374	0.89	0.7
EN-RAGE	NM_005621	134	282	0.47	45	46	0.97	0.7
Ferritin	NM_000146	1823	2526	0.70	218	294	0.74	0.7
Activin A	NM_002192	7158	15597	0.46	1738	1783	0.97	0.7
EMAP-II	NM_012155	1122	2031	0.55	203	230	0.88	0.7
TRA-1-81	NM_001018111	1129	1688	0.70	280	391	0.72	0.7
CCR1	NM_001295	191	232	0.82	39	66	0.59	0.7
Artemin	NM_057160	365	809	0.45	172	179	0.96	0.7
CRTH-2	NM_004778	794	1245	0.64	128	170	0.75	0.7
GDNF	NM_000514	368	414	0.89	53	106	0.50	0.7
Eotaxin / CCL11	NM_002986	366	697	0.52	99	116	0.85	0.7
GDF5	NM_000557	16039	23211	0.69	2335	3425	0.68	0.7
Endostatin	NM_030582	934	1452	0.64	127	175	0.73	0.7
CV-2 / Crossveinless-2	NM_005214	1285	1994	0.64	307	429	0.72	0.7
Dkk-3	NM_013253	671	1106	0.61	72	96	0.75	0.7
FGF-18	NM_033649/ NM_003862	2766	4492	0.62	379	514	0.74	0.7
CCL14 / HCC-1 / HCC-3	NM_032962	905	1930	0.47	135	153	0.88	0.7
ApoB100	NM_000483	77	112	0.70	58	89	0.65	0.7
CCR2	NM_000647	186	219	0.85	30	61	0.49	0.7
Frizzled-7	NM_003507	199	287	0.69	93	144	0.65	0.7
ApoB	NM_000384	341	624	0.50	163	195	0.84	0.7
IL-1 F9 / IL-1 H1	NM_019618	2212	2584	0.86	162	349	0.46	0.7
DcR3 / TNFRSF6B	NM_032945	296	482	0.61	77	111	0.70	0.7
RET	NM_020975	221	265	0.80	69	141	0.49	0.6
GDF9	NM_005260	4795	5378	0.89	1418	3632	0.39	0.6
VDUP-1	NM_006472	5474	14895	0.40	8194	9355	0.88	0.6
Angiopoietin-1	NM_001146. NM_139290	600	1163	0.52	255	337	0.76	0.6
AMPKa1	NM_006251	116	370	0.30	83	86	0.96	0.6
CD23	NM_002002	90	152	0.60	68	103	0.66	0.6
DR3 / TNFRSF25	NM_148965	208	596	0.35	168	189	0.89	0.6
CLC	NM_001828	182	459	0.40	259	308	0.84	0.6
BMP-6	NM_001718	184	487	0.38	114	137	0.83	0.6
Activin RII A/B	NM_001106	130	194	0.67	17	33	0.51	0.6
FADD	NM_003824	449	599	0.75	23	54	0.42	0.6
Fas Ligand	NM_000639	425	499	0.85	43	139	0.31	0.6
Axl	NM_021913	288	566	0.51	56	89	0.63	0.6
CD24	NM_013230	66	198	0.30	134	160	0.84	0.6
CCR6	NM_031409	74	107	0.69	27	63	0.42	0.6
AgRP	NM_001138	827	2929	0.28	308	373	0.83	0.6
BAF57	NM_003079	23	56	0.40	53	79	0.67	0.5

Activin RIB / ALK-4	NM_020328	205	382	0.54	44	94	0.47	0.5
ALCAM	NM_001627	222	277	0.80	42	211	0.20	0.5
BMP-7	NM_001719	530	1602	0.33	279	431	0.65	0.5
BAFF R / TNFRSF13C	NM_052945	168	341	0.49	62	129	0.48	0.5
B7-1 /CD80	NM_005191	264	704	0.37	68	122	0.56	0.5
Angiopoietin-like 2	NM_012098	1933	6205	0.31	238	405	0.59	0.4
ApoC2	NM_001645	21	113	0.20	62	100	0.62	0.4
Beta 2M	NM_004048	1	40	0.00	80	114	0.70	0.4
TAF4	NM_003185	1	78	0.00	31	48	0.64	0.3
Calbindin D	NM_004929	1	52	0.00	59	102	0.58	0.3
RIP1	NM_003804	1	42	0.00	50	103	0.49	0.2
pro-Glucagon	NM_002054	1	56	0.00	33	85	0.39	0.2
IL-36RN	NM_173170	1	56	0.00	28	80	0.35	0.2
FGF R3	NM_022965	1	81	0.01	24	105	0.23	0.1

Protein analysis of cEV/PDGF-EV samples performed in 2 independent experiments (in blue PDGF-EV, in rose cEVs, in yellow RQ of the first and second experiment). Only proteins consistently up-regulated or down-regulated in PDGF-EVs, or proteins expressed with RQ more than 0.5 and less than 2 in both experiments are presented. In bold – up-regulated in PDGF-EVs proteins, in italic – down-regulated in PDGF-EVs proteins.

Supplementary figure S2. Protein pattern of cEV and PDGF-EVs.

a

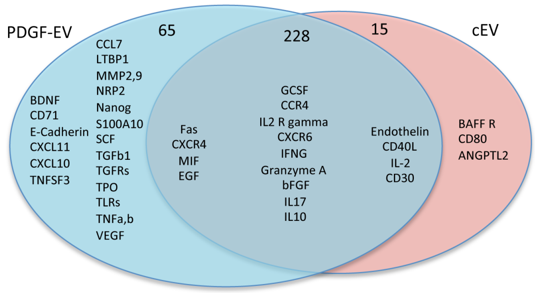

b

PDGF-EV specific Pathways	Entities found	p value
Immune System	46	1.89E-6
Cytokine Signaling in Immune system	30	4.01E-10
Signaling by Interleukins	20	8.37E-8
Signaling by Receptor Tyrosine Kinases	17	1.11E-5
Extracellular matrix organization	15	8.37E-7
Peptide ligand-binding receptors	11	6.39E-6
TNFR2 non-canonical NF-kB pathway	10	1.73E-7
Interleukin-4 and Interleukin-13 signaling	9	3.41E-6
Platelet degranulation	9	1.06E-5
Response to elevated platelet cytosolic Ca2+	9	1.43E-5
TNFs bind their physiological receptors	8	1.63E-9
Chemokine receptors bind chemokines	8	5.96E-8
Activation of Matrix Metalloproteinases	7	8.22E-8
Collagen degradation	7	6.47E-6
Interleukin-10 signaling	6	1,00E-05
VEGF ligand-receptor interactions	4	1.95E-6

Common Pathways	Entities found	p value
Immune System	162	1.75E-8
Cytokine Signaling in Immune system	103	1.11E-16
Signaling by Interleukins	84	1.11E-16
MAPK family signaling cascades	43	6.23E-10
MAPK1/MAPK3 signaling	41	1.16E-10
Extracellular matrix organization	41	5.47E-9
RAF/MAP kinase cascade	37	6.94E-9
Peptide ligand-binding receptors	31	7.26E-9
Interleukin-4 and Interleukin-13 signaling	28	3.22E-12
PI5P, PP2A and IER3 Regulate PI3K/AKT Signaling	26	5.22E-11
Negative regulation of the PI3K/AKT network	26	1.96E-10
Degradation of the extracellular matrix	24	1.67E-7
PI3K/AKT Signaling in Cancer	23	4.65E-9
Constitutive Signaling by Aberrant PI3K in Cancer	22	1.12E-10
Chemokine receptors bind chemokines	20	7.13E-13
Interleukin-10 signaling	18	1.93E-11
Interleukin-2 family signaling	16	1.34E-9
Insulin receptor signalling cascade	16	6.9E-8
Signaling by activated point mutants of FGFR1	9	1.16E-8

cEV specific Pathways	Entities found	p value
Signaling by FGFR3 in disease	3	2.86E-6
Constitutive Signaling by Aberrant PI3K in Cancer	3	9.83E-5
Phospholipase C-mediated cascade; FGFR3	2	1.23E-4
FGFR3 ligand binding and activation	2	1.23E-4
PI3K/AKT Signaling in Cancer	3	2.33E-4
PI5P, PP2A and IER3 Regulate PI3K/AKT Signaling	3	2.46E-4
Diseases of signal transduction	4	8.67E-4
PI3K Cascade	2	1.54E-3

Supplementary figure S2. Protein pattern of cEV and PDGF-EVs. (a) proteins relevant for inflammation: PDGF-EVs - in blue, cEVs - in rose, in the middle – equally expressed proteins; (b) overrepresented signaling pathways found by bioinformatic analysis of up-regulated protein sets for both types of EVs.

Supplementary table 2. Expression of microRNA in cEVs and PDGF-EVs.

PDGF-EV		expressed in both cEVs and PDGF-EVs					cEV	
Target Name	C_T Mean	Target Name	C_T Mean PDGF-EV	C_T Mean cEV	RQ	P-Value	Target Name	C_T Mean
**hsa-miR-802**	**16.6**	**hsa-miR-1226-5p**	**29.8**	**29.4**	**0.5**	**0.005**	**hsa-miR-1238**	**13.5**
**hsa-miR-765**	**18.5**	**hsa-miR-203a-3p**	**30.4**	**31.5**	**2.0**	**0.017**	**hsa-miR-1204**	**14.9**
**hsa-miR-380-3p**	**18.8**	**hsa-miR-99a-3p**	**30.6**	**33.6**	**5.9**	**0.030**	**hsa-miR-641**	**17.5**
**hsa-miR-551b**	**20.4**	**hsa-miR-125b**	**23.0**	**23.8**	**2.4**	**0.031**	**hsa-miR-659**	**19.0**
**hsa-miR-872**	**23.8**	**hsa-miR-195**	**27.7**	**28.3**	**2.4**	**0.034**	**hsa-miR-1253**	**23.5**
**hsa-miR-211**	**26.4**	**hsa-miR-1225-3p**	**29.1**	**27.8**	**0.3**	**0.050**	**hsa-miR-376b**	**24.8**
**hsa-miR-567**	**27.9**	dme-miR-7	33.4	34.9	1.2	1.000	**hsa-miR-556-3p**	**25.9**
**hsa-miR-208b**	**30.1**	hsa-let-7a	25.9	26.1	1.2	0.985	**hsa-miR-1272**	**26.0**
**hsa-miR-708-3p**	**30.2**	hsa-let-7b	21.7	21.2	0.7	0.758	**hsa-miR-205**	**27.0**
**hsa-miR-1296**	**30.7**	hsa-let-7b#	25.8	29.4	13.9	1.000	**hsa-miR-330-5p**	**28.8**
hsa-let-7e#	34.1	hsa-let-7c	27.2	27.7	1.5	0.386	**hsa-miR-183-3p**	**30.0**
hsa-let-7f-2#	32.0	hsa-let-7d	26.4	26.7	1.2	0.753	**hsa-miR-500**	**30.6**
hsa-miR-122#	31.5	hsa-let-7e	22.7	19.4	0.1	0.359	hsa-miR-1	32.9
hsa-miR-1269	32.7	hsa-let-7f	28.0	29.7	3.4	0.632	hsa-miR-1249	33.4
hsa-miR-1276	33.9	hsa-let-7g	27.3	27.3	1.1	0.909	hsa-miR-1256	34.9
hsa-miR-129	33.4	hsa-let-7i#	32.0	32.1	0.8	0.511	hsa-miR-130a#	33.4
hsa-miR-133b	34.3	hsa-miR-100	21.9	22.4	1.5	0.719	hsa-miR-132#	34.1
hsa-miR-147b	33.5	hsa-miR-100#	33.9	32.8	0.5	1.000	hsa-miR-138-2#	34.1
hsa-miR-154	32.0	hsa-miR-101	33.4	32.8	0.8	0.678	hsa-miR-142-5p	31.8
hsa-miR-186#	32.8	hsa-miR-103	28.8	28.9	1.1	0.529	hsa-miR-144	31.6
hsa-miR-202	33.1	hsa-miR-106a	24.8	24.1	0.6	0.470	hsa-miR-183	33.7
hsa-miR-216b	34.3	hsa-miR-106b	27.5	27.9	1.4	0.727	hsa-miR-192#	34.7
hsa-miR-221#	33.0	hsa-miR-106b#	30.9	31.1	0.8	0.365	hsa-miR-200a#	33.6
hsa-miR-23b#	34.4	hsa-miR-107	33.1	31.8	0.4	0.677	hsa-miR-24-1#	34.5
hsa-miR-296-3p	34.2	hsa-miR-10a	27.0	27.1	1.1	0.815	hsa-miR-32	33.6
hsa-miR-338-5P	33.7	hsa-miR-10a#	29.6	31.6	3.1	0.265	hsa-miR-383	31.4
hsa-miR-34b	31.8	hsa-miR-10b	25.6	26.0	1.4	0.606	hsa-miR-429	32.0
hsa-miR-373	34.8	hsa-miR-10b#	26.2	27.2	1.4	0.948	hsa-miR-453	35.0
hsa-miR-381	33.5	hsa-miR-1179	33.4	32.3	0.4	0.931	hsa-miR-483-3p	31.0
hsa-miR-432#	32.8	hsa-miR-1180	30.7	30.7	0.9	0.656	hsa-miR-487a	33.0
hsa-miR-502	31.3	hsa-miR-1183	34.4	32.5	0.1	1.000	hsa-miR-488	33.6
hsa-miR-511	33.3	hsa-miR-1208	33.3	32.1	0.3	0.498	hsa-miR-508	34.9
hsa-miR-518a-5p	34.6	hsa-miR-122	32.0	30.6	0.3	1.000	hsa-miR-512-5p	31.7
hsa-miR-525-3p	32.4	hsa-miR-1227	32.3	31.9	0.6	0.483	hsa-miR-515-5p	33.9
hsa-miR-548c-5p	33.7	hsa-miR-1228#	23.9	30.1	71.4	1.000	hsa-miR-516-3p	32.7
hsa-miR-548I	31.5	hsa-miR-1233	26.0	26.8	1.6	0.801	hsa-miR-517c	32.8
hsa-miR-550	33.4	hsa-miR-1244	32.3	32.3	0.8	0.878	hsa-miR-519e#	32.8
hsa-miR-562	34.8	hsa-miR-1247	25.3	24.7	0.5	0.900	hsa-miR-521	31.7
hsa-miR-575	33.8	hsa-miR-1254	29.4	29.5	0.8	0.474	hsa-miR-541	31.3
hsa-miR-579	34.5	hsa-miR-1255B	32.6	33.0	0.9	0.688	hsa-miR-545	33.2
hsa-miR-581	34.4	hsa-miR-125a-3p	31.4	30.2	0.7	0.576	hsa-miR-592	32.9
hsa-miR-582-5p	34.8	hsa-miR-125a-5p	25.1	25.4	1.3	0.641	hsa-miR-595	32.1
hsa-miR-604	34.4	hsa-miR-125b-1#	28.8	30.0	1.6	0.530	hsa-miR-875-5p	33.7
hsa-miR-614	33.0	hsa-miR-125b-2#	27.6	29.0	1.9	0.328	hsa-miR-876-5p	34.5
hsa-miR-621	34.5	hsa-miR-126	27.2	24.5	0.2	0.327	hsa-miR-892b	31.4
hsa-miR-643	32.4	hsa-miR-126#	32.4	28.7	0.1	0.404	hsa-miR-9#	32.9
hsa-miR-92a-1#	34.6	hsa-miR-1260	25.1	27.5	3.6	0.398	hsa-miR-937	34.4
hsa-miR-943	33.7	hsa-miR-1262	15.7	18.2	2.8	0.972	hsa-miR-938	34.4
hsa-miR-944	33.6	hsa-miR-1267	34.0	31.1	0.1	0.439		
		hsa-miR-127	26.3	27.0	1.7	0.259		
		hsa-miR-1270	31.0	31.9	1.3	0.528		
		hsa-miR-1271	26.5	27.8	1.7	0.451		
		hsa-miR-1274A	17.4	18.2	1.3	0.771		
		hsa-miR-1274B	16.5	17.2	1.1	0.673		

hsa-miR-1275	27.7	28.6	1.3	0.911
hsa-miR-1285	27.5	30.3	4.9	0.227
hsa-miR-128a	30.6	31.0	1.1	0.619
hsa-miR-1290	25.5	24.2	0.3	0.348
hsa-miR-1291	29.1	29.7	1.1	0.807
hsa-miR-1298	25.7	20.6	0.0	0.500
hsa-miR-1300	27.8	25.9	0.2	0.420
hsa-miR-1303	29.7	31.3	2.8	0.366
hsa-miR-1305	22.7	30.2	109.5	0.479
hsa-miR-130a	28.8	28.7	1.0	0.349
hsa-miR-130b	28.2	28.4	1.9	0.206
hsa-miR-130b#	29.2	28.6	0.6	0.471
hsa-miR-132	25.6	26.1	1.4	0.420
hsa-miR-133a	29.2	28.6	0.7	0.437
hsa-miR-135b	31.8	29.5	0.3	0.776
hsa-miR-135b#	31.2	30.7	1.0	1.000
hsa-miR-136	30.7	28.4	0.3	0.649
hsa-miR-136#	29.4	30.1	1.1	0.700
hsa-miR-138	26.0	27.0	2.0	0.271
hsa-miR-139-3p	33.1	29.7	0.1	0.185
hsa-miR-139-5p	30.7	28.6	0.2	0.329
hsa-miR-140-3p	30.8	29.9	0.5	0.500
hsa-miR-141	33.8	33.0	1.0	1.000
hsa-miR-142-3p	32.6	29.1	0.1	0.320
hsa-miR-143	27.5	27.6	1.1	0.729
hsa-miR-144#	34.7	30.4	0.0	1.000
hsa-miR-145	22.6	23.2	1.6	0.374
hsa-miR-145#	31.0	32.5	2.1	0.393
hsa-miR-146a	23.3	23.1	0.9	0.839
hsa-miR-146b	24.5	24.0	0.8	0.637
hsa-miR-146b-3p	33.5	30.6	0.3	1.000
hsa-miR-148a	29.1	30.3	2.4	0.295
hsa-miR-148a#	32.9	32.8	0.8	0.718
hsa-miR-148b	30.3	28.6	0.6	0.557
hsa-miR-148b#	32.6	32.4	0.6	0.561
hsa-miR-149	25.2	25.8	1.6	0.497
hsa-miR-150	28.0	25.1	0.1	0.280
hsa-miR-151-3p	23.8	24.5	1.2	0.757
hsa-miR-151-5P	25.6	26.6	1.3	0.577
hsa-miR-152	26.3	26.5	1.2	0.479
hsa-miR-154#	34.5	33.4	0.4	1.000
hsa-miR-155	24.9	24.5	0.8	0.570
hsa-miR-15a	29.1	27.6	0.3	0.238
hsa-miR-15a#	29.5	33.4	11.4	0.283
hsa-miR-15b	28.0	28.0	1.0	0.766
hsa-miR-15b#	28.1	27.6	0.5	0.911
hsa-miR-16	23.9	23.4	0.7	0.525
hsa-miR-16-1#	30.9	31.8	1.3	0.533
hsa-miR-16-2#	29.5	29.3	0.3	1.000
hsa-miR-17	25.4	24.9	0.7	0.558
hsa-miR-17#	33.0	33.8	0.8	1.000
hsa-miR-181a	27.2	27.4	1.2	0.860
hsa-miR-181a-2#	29.9	29.9	0.7	0.857
hsa-miR-181c	33.5	32.2	0.5	0.552
hsa-miR-181c#	31.9	30.5	0.3	0.958
hsa-miR-182	33.6	30.1	0.2	1.000
hsa-miR-1825	31.2	34.4	7.2	0.504
hsa-miR-184	31.3	30.4	0.5	0.602

	hsa-miR-185	29.9	31.3	2.0	0.358	
	hsa-miR-186	24.5	24.5	1.0	0.749	
	hsa-miR-18a	30.5	31.1	1.1	0.726	
	hsa-miR-18a#	31.7	31.5	0.6	0.597	
	hsa-miR-18b	32.6	29.0	0.1	0.427	
	hsa-miR-190	34.2	33.5	0.4	0.533	
	hsa-miR-190b	20.9	28.7	155.5	0.397	
	hsa-miR-191	22.4	21.2	0.4	0.337	
	hsa-miR-191#	31.3	30.2	0.4	0.411	
	hsa-miR-192	31.7	30.4	0.4	0.608	
	hsa-miR-193a-3p	29.1	31.4	3.6	0.102	
	hsa-miR-193a-5p	26.4	27.2	1.7	0.510	
	hsa-miR-193b	21.1	21.0	1.0	0.859	
	hsa-miR-193b#	28.5	29.0	1.0	0.753	
	hsa-miR-194	31.9	31.1	0.6	0.516	
	hsa-miR-196b	28.5	28.4	1.0	0.989	
	hsa-miR-197	23.4	23.4	1.0	0.959	
	hsa-miR-198	32.6	33.5	2.0	0.655	
	hsa-miR-199a	30.5	32.4	4.0	0.254	
	hsa-miR-199a-3p	24.5	25.0	1.4	0.576	
	hsa-miR-199b	29.6	29.0	0.7	0.420	
	hsa-miR-19a	29.8	30.0	1.1	0.825	
	hsa-miR-19b	24.0	24.1	1.1	0.693	
	hsa-miR-19b-1#	31.4	31.8	0.9	0.669	
	hsa-miR-200a	31.8	27.4	0.1	0.440	
	hsa-miR-200b	30.3	28.6	0.6	1.000	
	hsa-miR-200c	30.4	28.7	0.3	0.213	
	hsa-miR-204	27.6	28.0	1.4	0.809	
	hsa-miR-206	33.2	33.3	0.9	0.830	
	hsa-miR-20a	25.4	25.3	1.0	0.874	
	hsa-miR-20a#	32.8	33.6	1.8	1.000	
	hsa-miR-20b	30.4	29.7	0.7	0.396	
	hsa-miR-21	23.0	23.4	1.4	0.332	
	hsa-miR-21#	29.4	30.2	1.2	0.802	
	hsa-miR-210	23.4	24.5	1.5	0.730	
	hsa-miR-212	26.7	27.6	2.0	0.756	
	hsa-miR-213	32.0	32.7	1.1	0.992	
	hsa-miR-214	25.8	23.4	0.2	0.985	
	hsa-miR-214#	27.5	29.4	2.7	0.312	
	hsa-miR-215	29.1	31.5	3.7	0.184	
	hsa-miR-216a	30.3	31.7	2.2	1.000	
	hsa-miR-218	23.7	25.4	2.3	0.212	
	hsa-miR-218-2#	32.8	33.7	2.6	1.000	
	hsa-miR-219	26.7	27.9	2.0	1.000	
	hsa-miR-219-2-3p	20.6	23.3	2.7	1.000	
	hsa-miR-22	24.1	24.7	1.7	0.887	
	hsa-miR-22#	27.0	29.5	4.1	0.208	
	hsa-miR-221	24.7	25.8	2.1	0.379	
	hsa-miR-222	19.0	20.0	2.1	0.232	
	hsa-miR-222#	24.7	26.9	3.1	0.573	
	hsa-miR-223	27.9	25.3	0.2	0.353	
	hsa-miR-223#	33.4	30.5	0.1	0.161	
	hsa-miR-224	27.5	27.8	1.3	0.399	
	hsa-miR-23a	28.4	30.3	3.8	0.164	
	hsa-miR-24	19.5	19.6	1.1	0.763	
	hsa-miR-24-2#	29.5	31.4	3.3	0.299	
	hsa-miR-25	29.2	29.3	1.1	0.752	
	hsa-miR-25#	30.4	31.8	1.6	0.559	

hsa-miR-26a	25.9	26.0	1.1	0.852
hsa-miR-26a-1#	30.8	31.1	0.8	0.751
hsa-miR-26b	28.8	29.6	1.8	0.835
hsa-miR-26b#	31.4	32.5	1.5	0.908
hsa-miR-27a	28.0	28.2	1.2	0.438
hsa-miR-27a#	27.7	28.8	1.5	0.962
hsa-miR-27b	28.0	28.8	1.9	0.406
hsa-miR-27b#	29.4	27.6	0.3	0.461
hsa-miR-28	28.5	28.7	1.2	0.498
hsa-miR-28-3p	25.4	25.8	1.4	0.323
hsa-miR-296	28.2	30.4	4.0	0.249
hsa-miR-299-3p	30.8	28.8	0.2	1.000
hsa-miR-299-5p	25.0	21.9	0.1	0.502
hsa-miR-29a	23.0	23.5	1.4	0.462
hsa-miR-29a#	29.6	31.5	2.7	0.476
hsa-miR-29b	30.9	30.4	1.3	0.464
hsa-miR-29b-1#	31.7	33.2	1.1	1.000
hsa-miR-29b-2#	33.4	34.0	1.1	1.000
hsa-miR-29c	33.3	34.5	2.3	0.312
hsa-miR-301	29.1	29.1	1.0	0.746
hsa-miR-301b	35.0	34.0	0.6	1.000
hsa-miR-302a	26.5	26.5	2.6	1.000
hsa-miR-302d	33.5	32.1	0.5	1.000
hsa-miR-30a-3p	23.3	23.2	0.7	0.500
hsa-miR-30a-5p	25.2	25.8	1.0	0.990
hsa-miR-30b	25.8	26.0	1.2	0.566
hsa-miR-30c	25.4	25.7	1.3	0.568
hsa-miR-30d	27.6	26.4	0.3	0.427
hsa-miR-30d#	29.0	29.0	0.7	0.900
hsa-miR-30e-3p	23.3	23.3	0.7	0.448
hsa-miR-31	22.0	22.4	1.4	0.885
hsa-miR-31#	22.5	24.0	2.0	0.931
hsa-miR-320	23.0	22.7	0.8	0.596
hsa-miR-320B	29.1	29.6	1.0	0.965
hsa-miR-323-3p	28.9	28.4	0.7	0.907
hsa-miR-324-3p	28.1	27.6	0.7	0.842
hsa-miR-324-5p	29.9	30.4	1.6	0.493
hsa-miR-326	33.0	32.5	1.3	0.440
hsa-miR-328	28.1	28.7	1.6	0.575
hsa-miR-329	26.6	18.6	0.0	1.000
hsa-miR-330	29.6	30.0	1.0	0.773
hsa-miR-331	24.5	24.7	1.2	0.838
hsa-miR-331-5p	27.7	28.7	3.5	0.342
hsa-miR-335	28.0	29.6	3.1	0.132
hsa-miR-335#	27.7	28.7	1.4	0.701
hsa-miR-337-3p	29.6	34.9	30.6	0.245
hsa-miR-337-5p	30.4	28.9	0.6	0.429
hsa-miR-338-3p	33.5	32.5	0.6	0.564
hsa-miR-339-3p	28.4	27.8	0.7	0.472
hsa-miR-339-5p	28.7	30.0	2.5	0.212
hsa-miR-33a	34.4	33.7	0.5	0.612
hsa-miR-33a#	31.0	32.8	2.4	0.363
hsa-miR-33b	32.0	29.9	0.5	0.679
hsa-miR-340	32.1	32.5	1.1	0.987
hsa-miR-340#	32.2	31.2	0.4	0.545
hsa-miR-342-3p	22.0	21.5	0.8	0.948
hsa-miR-342-5p	30.3	26.4	0.3	1.000
hsa-miR-345	28.1	28.3	1.2	0.553

	hsa-miR-34a	27.4	28.0	1.6	0.220	
	hsa-miR-34a#	24.9	25.8	1.3	0.483	
	hsa-miR-34b	26.2	27.5	1.7	0.280	
	hsa-miR-34c	31.5	31.1	0.9	0.633	
	hsa-miR-361	30.0	30.1	2.0	0.245	
	hsa-miR-361-3p	31.3	30.1	0.5	1.000	
	hsa-miR-362	28.5	29.2	1.2	0.867	
	hsa-miR-362-3p	32.1	32.5	1.4	0.359	
	hsa-miR-365	26.7	30.0	11.8	0.451	
	hsa-miR-369-3p	33.8	33.4	2.0	1.000	
	hsa-miR-369-5p	34.4	33.7	2.5	1.000	
	hsa-miR-370	26.9	27.0	1.1	0.455	
	hsa-miR-374	28.4	27.2	0.5	0.722	
	hsa-miR-375	33.1	32.5	1.0	0.874	
	hsa-miR-376a	26.7	26.6	1.0	0.804	
	hsa-miR-376a#	32.8	30.6	0.2	0.501	
	hsa-miR-376c	26.2	26.1	0.9	0.776	
	hsa-miR-377#	34.0	33.8	0.4	1.000	
	hsa-miR-378	29.0	29.0	0.7	0.708	
	hsa-miR-378	33.0	33.9	2.0	1.000	
	hsa-miR-380-5p	31.1	33.2	3.0	0.347	
	hsa-miR-382	24.8	24.7	1.0	0.619	
	hsa-miR-384	25.6	25.7	2.8	1.000	
	hsa-miR-409-3p	20.9	21.4	1.0	0.932	
	hsa-miR-409-5p	32.6	34.7	7.7	0.052	
	hsa-miR-410	29.9	30.1	1.2	0.860	
	hsa-miR-411	28.5	29.1	1.5	0.785	
	hsa-miR-411#	32.4	32.6	0.8	0.869	
	hsa-miR-412	32.3	34.1	2.2	0.811	
	hsa-miR-422a	32.8	28.2	0.1	1.000	
	hsa-miR-423-5p	30.7	30.6	1.0	0.323	
	hsa-miR-424	30.8	32.4	3.4	0.192	
	hsa-miR-424#	28.1	29.5	1.9	0.096	
	hsa-miR-425-5p	26.7	27.1	1.3	0.949	
	hsa-miR-425#	30.3	28.6	0.2	0.200	
	hsa-miR-431	28.0	28.0	1.1	0.687	
	hsa-miR-432	25.7	24.9	0.4	0.246	
	hsa-miR-433	31.8	29.7	0.2	0.286	
	hsa-miR-449	32.2	33.3	3.9	0.456	
	hsa-miR-449b	35.0	33.4	0.4	1.000	
	hsa-miR-450a	32.3	31.5	0.7	1.000	
	hsa-miR-450b-3p	32.0	30.8	1.1	1.000	
	hsa-miR-450b-5p	34.6	34.5	1.6	0.328	
	hsa-miR-452	28.0	28.9	1.4	0.783	
	hsa-miR-454	27.4	26.7	0.6	0.323	
	hsa-miR-454#	32.1	33.5	1.9	0.866	
	hsa-miR-455	30.8	30.7	0.9	0.844	
	hsa-miR-455-3p	30.5	29.0	0.7	0.833	
	hsa-miR-483-5p	31.0	32.9	2.8	0.355	
	hsa-miR-484	22.3	21.9	0.8	0.893	
	hsa-miR-485-3p	30.3	30.0	1.0	0.879	
	hsa-miR-485-5p	29.2	30.6	2.0	0.833	
	hsa-miR-486	32.8	29.7	0.1	0.209	
	hsa-miR-487b	29.1	30.1	1.5	0.717	
	hsa-miR-489	32.9	31.7	0.3	0.412	
	hsa-miR-492	31.8	31.8	2.2	0.587	
	hsa-miR-493	29.0	30.4	2.0	0.372	
	hsa-miR-494	28.8	28.3	0.7	0.779	

	hsa-miR-497	31.0	32.9	3.4	0.606	
	hsa-miR-500	31.3	33.2	2.8	0.313	
	hsa-miR-501	28.4	29.9	2.1	0.487	
	hsa-miR-501-3p	31.7	30.7	0.5	0.621	
	hsa-miR-502-3p	31.9	34.5	3.7	0.364	
	hsa-miR-503	30.6	30.0	0.5	0.398	
	hsa-miR-505	30.2	32.4	8.2	1.000	
	hsa-miR-505#	31.4	31.5	0.8	0.516	
	hsa-miR-512-3p	34.0	33.9	1.3	0.518	
	hsa-miR-513-5p	26.2	31.0	51.3	1.000	
	hsa-miR-517a	25.0	24.5	0.6	0.423	
	hsa-miR-518a-3p	24.6	27.8	16.7	0.509	
	hsa-miR-518b	26.1	24.7	2.0	1.000	
	hsa-miR-518d	28.5	29.6	2.4	0.609	
	hsa-miR-518f	25.1	29.5	30.1	0.576	
	hsa-miR-519a	24.8	23.8	1.8	0.595	
	hsa-miR-519b-3p	21.6	32.0	675.1	0.498	
	hsa-miR-520a	33.5	21.3	0.0	1.000	
	hsa-miR-520a#	15.3	19.2	26.0	1.000	
	hsa-miR-520b	28.8	31.6	8.7	1.000	
	hsa-miR-520c-3p	32.9	34.9	4.6	0.335	
	hsa-miR-520D-3P	27.4	27.4	0.7	0.881	
	hsa-miR-520d-5p	29.1	31.2	4.7	0.605	
	hsa-miR-520f	28.2	28.6	3.7	1.000	
	hsa-miR-520g	25.8	20.8	0.0	1.000	
	hsa-miR-523	24.5	23.5	1.4	1.000	
	hsa-miR-532	26.1	26.6	1.6	0.323	
	hsa-miR-532-3p	26.7	27.1	1.5	0.477	
	hsa-miR-539	27.4	27.3	1.0	0.991	
	hsa-miR-541#	32.9	28.1	0.0	1.000	
	hsa-miR-542-3p	32.1	33.9	3.1	0.509	
	hsa-miR-542-5p	30.4	31.4	1.4	0.816	
	hsa-miR-543	29.7	30.5	1.2	0.735	
	hsa-miR-545#	30.2	33.4	4.4	0.507	
	hsa-miR-548a	30.8	32.6	4.0	0.266	
	hsa-miR-548b	30.5	26.3	0.1	1.000	
	hsa-miR-548c	30.8	30.6	1.3	0.500	
	hsa-miR-548d-5p	30.9	32.3	1.1	1.000	
	hsa-miR-549	32.5	33.7	2.1	0.251	
	hsa-miR-550	31.0	32.9	2.5	0.282	
	hsa-miR-551a	13.3	22.6	280.2	1.000	
	hsa-miR-561	18.3	28.3	1.901485	1.000	
	hsa-miR-564	32.2	33.7	1.8	0.682	
	hsa-miR-566	28.9	34.8	61.2	1.000	
	hsa-miR-570	33.3	31.9	1.0	1.000	
	hsa-miR-571	31.2	31.9	1.2	0.581	
	hsa-miR-572	30.3	31.0	1.0	0.980	
	hsa-miR-574-3p	22.8	22.0	0.6	0.418	
	hsa-miR-576-3p	33.4	33.5	1.2	0.596	
	hsa-miR-582-3p	29.3	32.5	11.2	1.000	
	hsa-miR-584	30.5	29.0	0.3	0.108	
	hsa-miR-589	30.1	29.6	0.5	0.294	
	hsa-miR-589	34.4	34.5	3.0	1.000	
	hsa-miR-590-3P	29.5	30.5	1.5	0.974	
	hsa-miR-590-5p	28.6	29.3	1.7	0.185	
	hsa-miR-593	32.3	34.0	3.0	0.456	
	hsa-miR-596	27.2	30.0	5.2	0.756	
	hsa-miR-597	31.9	32.2	1.4	1.000	

hsa-miR-598	29.2	28.2	0.6	0.559
hsa-miR-603	30.2	30.2	0.7	0.618
hsa-miR-605	34.0	31.0	0.1	0.968
hsa-miR-615-5p	32.4	33.8	3.4	1.000
hsa-miR-616	33.1	31.9	0.4	0.098
hsa-miR-616	30.8	31.1	0.9	0.664
hsa-miR-618	33.9	33.6	2.2	1.000
hsa-miR-622	32.4	30.4	0.3	1.000
hsa-miR-623	30.8	26.7	0.0	1.000
hsa-miR-625	34.1	32.2	0.3	0.993
hsa-miR-625#	25.8	26.1	0.9	0.984
hsa-miR-627	29.8	33.9	19.3	0.513
hsa-miR-628-3p	29.1	28.3	0.4	0.116
hsa-miR-628-5p	30.3	30.7	0.9	0.498
hsa-miR-629	29.3	28.2	0.3	0.364
hsa-miR-629	31.7	33.6	10.3	1.000
hsa-miR-636	32.6	31.3	1.0	0.807
hsa-miR-638	28.2	28.7	1.3	0.409
hsa-miR-639	26.3	26.9	1.1	0.473
hsa-miR-642	32.7	28.2	0.1	0.503
hsa-miR-649	32.6	21.2	0.0	1.000
hsa-miR-650	30.6	30.8	0.8	0.618
hsa-miR-652	34.0	31.1	0.1	0.076
hsa-miR-654	29.7	30.8	2.4	0.546
hsa-miR-654-3p	34.2	34.5	1.0	1.000
hsa-miR-655	29.8	30.6	1.9	0.290
hsa-miR-656	30.4	31.4	1.6	0.577
hsa-miR-657	32.6	34.1	3.9	1.000
hsa-miR-660	26.9	26.8	1.0	0.836
hsa-miR-661	32.2	31.4	0.5	0.571
hsa-miR-663B	26.5	28.3	2.5	0.607
hsa-miR-664	24.9	25.4	1.0	0.881
hsa-miR-665	27.6	31.1	3.2	1.000
hsa-miR-668	34.2	33.2	0.4	0.766
hsa-miR-671-3p	31.9	32.7	2.2	0.428
hsa-miR-672	34.6	30.1	0.0	1.000
hsa-miR-7-2#	34.0	33.0	0.3	1.000
hsa-miR-708	28.4	28.5	1.2	0.640
hsa-miR-720	19.4	20.6	1.6	0.720
hsa-miR-744	28.5	29.4	2.0	0.486
hsa-miR-744#	31.1	31.9	1.8	1.000
hsa-miR-758	31.5	33.1	2.7	0.206
hsa-miR-766	26.9	27.5	1.1	0.970
hsa-miR-769-5p	29.2	31.4	3.2	0.517
hsa-miR-770-5p	27.5	27.1	0.5	0.350
hsa-miR-874	29.6	30.5	2.0	0.276
hsa-miR-885-5p	31.6	29.2	0.1	0.273
hsa-miR-886-3p	26.6	27.6	2.1	0.853
hsa-miR-886-5p	23.5	23.8	1.2	0.923
hsa-miR-887	34.5	33.1	0.4	1.000
hsa-miR-888	32.3	30.3	0.7	1.000
hsa-miR-889	31.2	29.2	0.4	0.220
hsa-miR-891a	31.4	31.0	2.0	1.000
hsa-miR-9	30.9	24.8	0.0	0.423
hsa-miR-922	30.7	34.1	8.6	1.000
hsa-miR-92a	26.1	26.2	1.1	0.505
hsa-miR-92b#	31.6	31.7	0.5	0.552
hsa-miR-93#	26.2	26.6	0.9	0.936

	hsa-miR-935	30.1	32.1	3.0	0.564	
	hsa-miR-939	34.4	29.6	0.0	1.000	
	hsa-miR-941	31.1	30.1	0.5	1.000	
	hsa-miR-942	30.4	30.8	1.0	0.801	
	hsa-miR-95	31.6	30.9	1.1	0.719	
	hsa-miR-98	22.4	28.4	68.3	0.391	
	hsa-miR-99a-5p	20.8	22.4	3.1	0.100	
	hsa-miR-99b	24.8	25.4	1.6	0.478	
	hsa-miR-99b#	28.2	26.3	0.2	0.246	
	RNU44	31.3	30.3	0.5	0.321	
	RNU48	27.4	26.3	0.5	0.345	
	U6 snRNA	22.8	20.1	0.1	0.266	

MicroRNAs detected in cEVs and PDGF-EVs (Ct<35): Ct, RQ (PDGF-EVs vs. cEVs) and p value are indicated. In blue column are present miRNAs expressed exclusively in PDGF-EVs. In rose column are present miRNAs exclusively expressed in cEVs. In bold – miRNAs selected for bioinformatic analysis.

Supplementary Figure S3. EV expression of MALAT1 or five selected miRNAs confirmed by Real-time PCR.

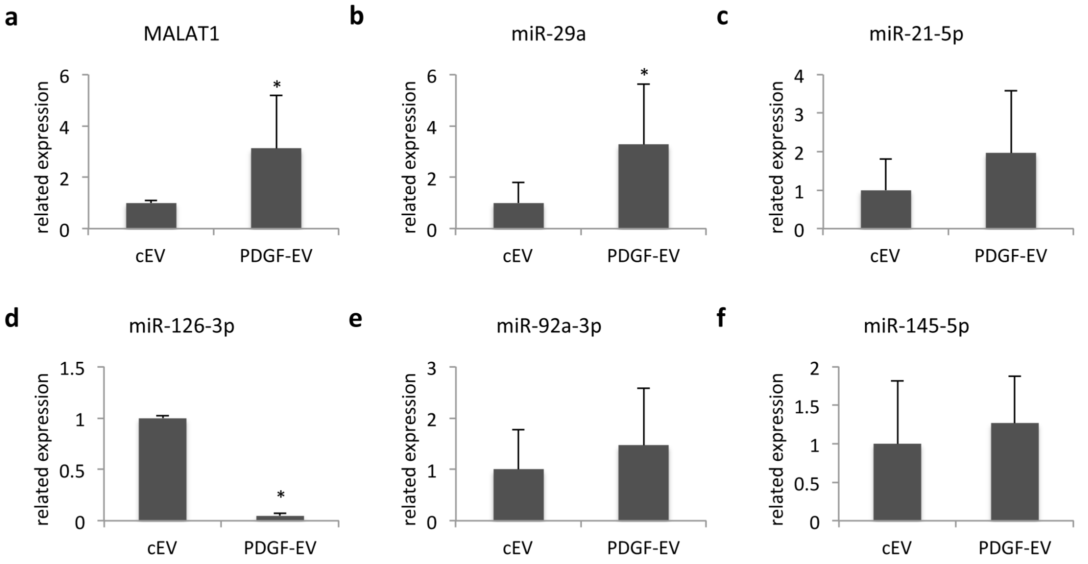

Supplementary Figure S3. EV expression of MALAT1 or five selected miRNAs confirmed by Real-time PCR. Diagrams of PCR analysis of RNA content of EVs: (a) expression of MALAT1; (b) expression of miR-29a; (c) expression of miR-21-5p; (d) expression of miR-126-3p; (e) expression of miR-92a-3p; (f) expression of miR-145-5p. * - $p<0.05$ vs. cEV, n=4.

Supplementary table 3. KEGG pathways overrepresented by cEV or PDGF-EV miRNAs.

PDGF-EVs			cEVs		
**KEGG pathway**	**#genes**	**#miRNAs**	**KEGG pathway**	**#genes**	**#miRNAs**
Hepatitis B	40	8	Proteoglycans in cancer	37	7
Prostate cancer	29	8	Hippo signaling pathway	28	7
Pathways in cancer	80	8	Lysine degradation	12	7
Epstein-Barr virus infection	47	8	Cell cycle	34	7
HTLV-I infection	53	8	Glioma	17	7
Insulin signaling pathway	33	8	Chronic myeloid leukemia	19	7
Estrogen signaling pathway	24	7	p53 signaling pathway	20	7
Chronic myeloid leukemia	20	7	Bladder cancer	13	7
FoxO signaling pathway	34	7	Small cell lung cancer	22	7
Melanoma	18	7	Pathways in cancer	63	7
Proteoglycans in cancer	48	6	Melanoma	18	7
Adherens junction	22	6	Hepatitis B	30	7
Hippo signaling pathway	37	6	RNA transport	32	7
Viral carcinogenesis	47	6	Transcriptional misregulation in cancer	28	7
**TGF-beta signaling pathway**	21	6	Endometrial cancer	13	7
Shigellosis	21	6	Acute myeloid leukemia	15	7
Cell cycle	35	6	Prostate cancer	22	7
p53 signaling pathway	21	6	HTLV-I infection	46	7
**TNF signaling pathway**	28	6	Non-small cell lung cancer	14	7
Bacterial invasion of epithelial cells	19	6	Pancreatic cancer	16	7
Progesterone-mediated oocyte maturation	24	6	Viral carcinogenesis	36	7
NOD-like receptor signaling pathway	17	6	Focal adhesion	38	7
Glioma	16	6	Ubiquitin mediated proteolysis	24	7
Endometrial cancer	14	6	Endocytosis	33	7
Small cell lung cancer	22	5	Thyroid cancer	13	6
Oocyte meiosis	29	4	Adherens junction	16	6
Long-term depression	14	4	Oocyte meiosis	25	6
Bladder cancer	14	4	Renal cell carcinoma	17	6
Fatty acid degradation	6	1	Epstein-Barr virus infection	35	6
			Protein processing in endoplasmic reticulum	29	6
			Central carbon metabolism in cancer	17	5
			Neurotrophin signaling pathway	24	5
			Bacterial invasion of epithelial cells	16	5
			Prion diseases	6	4
			Fatty acid metabolism	5	4
			Base excision repair	7	4
			Glycosaminoglycan biosynthesis - heparan sulfate / heparin	5	4
			Shigellosis	15	4
			Nucleotide excision repair	10	4
			Fatty acid biosynthesis	2	2
			Fatty acid elongation	2	2

Bioinformatic analysis of miRNAs carried by cEVs or PDGF-EVs performed by Diana tools mirPath V3 revealed statistically confirmed ($p<0.05$) KEGG pathways, which could be regulated by the selected miRNAs (see Supplementary Table S2, in bold): in blue column are present KEGG pathways overrepresented for PDGF-EV miRNAs, in rose column – KEGG pathways overrepresented for cEV miRNAs. Inflammation related pathways "TGF-beta signaling pathway" and "TNF signaling pathway" are present only in PDGF-EV group.
